# Reevaluating *Sirococcus*: synonymizing *Gnomoniopsis* and elucidating the life cycle of *S. daii*

**DOI:** 10.3897/imafungus.17.186049

**Published:** 2026-03-12

**Authors:** Ning Jiang, Nalin N. Wijayawardene, Han Xue, Yong Li

**Affiliations:** 1 Key Laboratory of Forest Protection of National Forestry and Grassland Administration, Ecology and Nature Conservation Institute, Chinese Academy of Forestry, Beijing, 100091, China Qujing Normal University Qujing China; 2 Center for Yunnan Plateau Biological Resources Protection and Utilization, Yunnan International Joint Laboratory of Fungal Sustainable Utilization in South and Southeast Asia, College of Biology and Food Engineering, Qujing Normal University, Qujing, Yunnan Province, 655011, China Ecology and Nature Conservation Institute, Chinese Academy of Forestry Beijing China

**Keywords:** *Castanea mollissima*, *
Diaporthales
*, generic synonymy, latent infection, life cycle, taxonomy

## Abstract

Historically, *Sirococcus* (established in 1855) and *Gnomoniopsis* (proposed in 1893) have been treated as distinct genera within *Gnomoniaceae*, primarily distinguished by the prevalence of their asexual and sexual morphs, respectively. However, recent molecular data have challenged this distinction. In this study, we re-evaluated the relationship between these two genera using a combined multi-locus phylogeny (ITS, *tef*1, and *tub*2) and morphological assessment. Phylogenetic analyses revealed that species of *Sirococcus* and *Gnomoniopsis* cluster within a single, robustly supported monophyletic lineage, sharing indistinguishable asexual characteristics. Consequently, we propose to synonymize *Gnomoniopsis* under *Sirococcus* following the principle of priority. Thirty-eight new combinations and a new species are proposed. Furthermore, we investigated the life cycle of *Sirococcus daii***comb. nov**., a severe pathogen causing nut rot of Chinese chestnut (*Castanea mollissima*), for which the teleomorph was previously unknown. Through extensive sampling, we discovered the teleomorph on overwintered leaf litter, confirming the saprobic phase of its life cycle. Additionally, the fungus was isolated from healthy female flowers, young nuts, and husks, suggesting a latent or endophytic infection strategy. Based on these findings, we reconstructed the complete life cycle of *S. daii*. This study not only resolves the taxonomic controversy surrounding *Sirococcus* and *Gnomoniopsis* but also provides crucial epidemiological insights into *S. daii*, facilitating the development of effective management strategies for chestnut nut rot.

## Introduction

The order *Diaporthales* (*Sordariomycetes*, *Ascomycota*) represents one of the ecologically most diverse and economically significant fungal lineages (Walker et al. 2012b; Senanayake et al. 2017, 2018; Fan et al. 2018; Jiang et al. 2019a, 2024, 2025b). Members of this order act as pivotal plant pathogens, innocuous endophytes, and saprobes involved in litter decomposition across a vast array of woody and herbaceous hosts (Jiang et al. 2021c, 2025c; Zhu et al. 2022; Du et al. 2025). Within this order, the family *Gnomoniaceae* has historically posed considerable taxonomic challenges (James et al. 2006; Sogonov et al. 2007, 2008; Rossman et al. 2008; Walker et al. 2010, 2012a; Mejía et al. 2011; Jiang et al. 2025a). Species in this family are notoriously pleomorphic, often exhibiting distinct sexual and asexual morphs that were historically treated as separate biological entities (Sogonov et al. 2008; Senanayake et al. 2018). This dual nomenclature system, coupled with the paucity of reliable morphological characters for generic delimitation, has led to a convoluted taxonomic history (Senanayake et al. 2018).

In the past two decades, the advent of multi-locus phylogenetics and the implementation of the “One Fungus, One Name” principle in the International Code of Nomenclature for algae, fungi, and plants (ICN) have necessitated a comprehensive re-evaluation of generic boundaries within the *Gnomoniaceae* (Hawksworth et al. 2009; Rossman 2014). Phylogenetic studies have increasingly exposed that many traditional genera, defined primarily by host association or a single morph state, are polyphyletic or paraphyletic (Rossman 2014; Rossman et al. 2015). Conversely, some genera previously separated by seemingly distinct morphological traits have been shown to represent different life stages of the same evolutionary lineage (Rossman et al. 2015).

The genus *Sirococcus* was established by Preuss (1855), typified by *Sirococcus conigenus* (= *Sirococcus strobilinus*, Sutton 1980; Cannon and Minter 1983). For over a century, *Sirococcus* has been widely recognized as a coelomycetous-typified genus (Preuss 1855; Rossman et al. 2008; Meyer et al. 2017). Its generic concept is firmly anchored in its asexual morphology: the production of pycnidial conidiomata, phialidic conidiogenous cells, and distinctive hyaline, fusiform to cylindrical conidia (Bahnweg et al. 2000; Smith et al. 2003; Broders and Boland 2011).

Ecologically, *Sirococcus* species are predominantly known as destructive pathogens of conifers. *S. conigenus* and *S. tsugae* are the causal agents of *Sirococcus* shoot blight, a disease that inflicts significant economic losses in silviculture and natural forests of *Cedrus*, *Picea*, *Pinus*, and *Tsuga* in North America and Europe (Bahnweg et al. 2000; Rossman et al. 2008; Broders and Boland 2011). Due to the conspicuous nature of these symptoms and the ubiquity of the asexual state on diseased tissues, the name *Sirococcus* has become deeply entrenched in forest pathology literature (Bahnweg et al. 2000; Smith et al. 2003; Konrad et al. 2007; Rossman et al. 2008; Broders and Boland 2011). However, the teleomorphs of *Sirococcus* species have not been reported, leading to a historical perception of the genus as an exclusively anamorphic lineage restricted to gymnosperms (Rossman et al. 2008; Senanayake et al. 2018).

In contrast, the genus *Gnomoniopsis* was introduced by Berlese in 1893 to accommodate species producing *Gnomonia*-like perithecia but differing in developing additional septa in ascospores (Sogonov et al. 2008; Walker et al. 2010; Crous et al. 2018). The type species, *Gnomoniopsis chamaemori*, is characterized by immersed perithecia with elongated beaks and small, hyaline ascospores (Sogonov et al. 2008; Crous et al. 2012). For a long time, *Gnomoniopsis* remained a relatively obscure genus until the resurrection of the name by Sogonov et al. (2008) to accommodate a specific clade within *Gnomoniaceae*.

Recently, *Gnomoniopsis* has gained substantial attention due to the global emergence of *Gnomoniopsis smithogilvyi* (syn. *G. castaneae*), the primary causal agent of sweet chestnut brown rot (Visentin et al. 2012; Shuttleworth et al. 2016; Lema et al. 2023). This pathogen causes severe nut rot and cankers on *Castanea* species, threatening chestnut production in Europe, North America, and Oceania (Shuttleworth and Guest 2017; Çakar 2024). While *Gnomoniopsis* is defined by its teleomorph, extensive studies on *G. smithogilvyi* and related species have revealed that they readily produce anamorphs in culture and in nature (Shuttleworth et al. 2016; Shuttleworth and Guest 2017). Crucially, these anamorphs are morphologically indistinguishable from *Sirococcus*: they produce identical pycnidial conidiomata, phialidic conidiogenous cells, and fusiform conidia (Rossman et al. 2008; Shuttleworth et al. 2016; Senanayake et al. 2018). This morphological congruence raises a fundamental question: are *Sirococcus* and *Gnomoniopsis* truly distinct genera, or do they represent the anamorphic and teleomorphic expressions of a single lineage?

Despite the morphological overlap of their asexual states, *Sirococcus* and *Gnomoniopsis* have been maintained as separate genera in most recent classifications (Senanayake et al. 2018). This separation appears to be largely artificial, driven by a historical emphasis on host preference—*Sirococcus* associated with conifers and *Gnomoniopsis* largely with broad-leaved trees (e.g., *Fagaceae*, *Rosaceae*, *Onagraceae*)—and the dominance of different morph states in their respective descriptions (Walker et al. 2010; Jiang et al. 2021b; Wang et al. 2022). However, host range is rarely a reliable delimiting character at the generic level in *Gnomoniaceae* (Sogonov et al. 2007, 2008; Walker et al. 2010).

Recent phylogenetic analyses utilizing multi-locus data have consistently recovered species of *Sirococcus* and *Gnomoniopsis* as a coherent, robustly supported clade (Rossman et al. 2008; Senanayake et al. 2018; Jiang et al. 2019b; Yang et al. 2020). Maintaining two generic names for a single monophyletic group contravenes the principles of phylogenetic systematics and creates unnecessary taxonomic instability. According to Article 11 of the ICN, the earliest legitimate name must take priority unless a later name is formally conserved. Since *Sirococcus* (1855) predates *Gnomoniopsis* (1893) by nearly four decades, and *Gnomoniopsis* is not a conserved name, synonymizing *Gnomoniopsis* under *Sirococcus* is not only scientifically justified by phylogenetic and morphological evidence but also nomenclaturally mandatory.

The urgency of resolving this taxonomic framework is underscored by the need to understand the biology of emerging pathogens within this complex. In China, the Chinese chestnut (*Castanea mollissima*) is a commercially vital crop. In recent years, a severe nut rot disease has been reported, caused by a pathogen previously identified as *Gnomoniopsis daii* (Jiang and Tian 2019; Jiang et al. 2021a). This pathogen causes significant yield reduction and post-harvest losses (Jiang and Tian 2019; Jiang et al. 2020).

To date, the understanding of *Gnomoniopsis daii* has been limited to its anamorph found on diseased leaves and in culture (Jiang and Tian 2019; Jiang et al. 2021a). The absence of knowledge regarding its teleomorph and its survival strategies during the winter has hampered the development of effective disease management protocols. In the life cycle of many *Gnomoniaceae* species, the sexual stage plays a critical role in overwintering on leaf litter and releasing ascospores as primary inoculum in the spring (Shuttleworth and Guest 2017). Therefore, clarifying the life cycle of *G. daii* is as critical as resolving its generic placement.

In this study, we undertook a comprehensive survey of *Gnomoniopsis*/*Sirococcus* associated with *Castanea* and other hosts in China. Our objectives were threefold: (1) to generate a robust multi-locus phylogeny to definitively resolve the relationship between *Sirococcus* and *Gnomoniopsis*; (2) to formally propose the synonymy of *Gnomoniopsis* under *Sirococcus* based on phylogenetic, morphological, and nomenclatural evidence, providing necessary new combinations; and (3) to elucidate the complete life cycle of the chestnut rot pathogen, *G. daii*.

## Materials and methods

### Surveys and sample collection

Since the first report of *Gnomoniopsis daii* on *Castanea mollissima* causing nut rot (Jiang and Tian 2019), comprehensive field surveys were conducted continuously from 2020 to 2025 to investigate the occurrence, distribution, and life cycle of this pathogen. Sampling was performed in major chestnut-growing regions in Beijing, Fujian, Guizhou, Hebei, Jiangxi, Shaanxi, and Shandong Provinces, China. To fully elucidate the life cycle and potential endophytic nature of the fungus, samples were collected from both symptomatic and asymptomatic tissues across different phenological stages of the host. Fresh leaves, young shoots, and nuts exhibiting typical rot or necrosis symptoms were collected during the growing season. Apparently healthy female flowers, young leaves, fruit husks, and developing nuts were randomly collected to screen for latent infections. To search for the teleomorph, leaf litter and decaying husks from the forest floor were collected specifically during the winter and early spring months. All samples were placed in separate paper bags, labeled with collection details, and transported to the laboratory. Samples were processed within 48 hours or stored at 4 °C until further examination.

### Fungal isolation

Pure culture isolations were performed using single-spore and tissue isolation techniques, depending on the nature of the sample. All obtained isolates were deposited in the China Forestry Culture Collection Center (CFCC).

Single-spore isolation: Fresh samples, particularly overwintered leaf litter, were examined under a stereomicroscope (Zeiss Discovery V8, Jena, Germany) for the presence of fungal fruiting bodies. Fungal structures suspected to belong to *Gnomoniaceae* were carefully removed using a sterile needle. Crushed perithecia were suspended in sterile distilled water to release asci and ascospores, while conidial masses from pycnidia were directly suspended in sterile water. The suspensions were streaked onto potato dextrose agar (PDA) plates. After incubation at 25 °C for 24 hours, germinating single spores were transferred to fresh PDA plates using a sterile needle under a stereomicroscope to establish pure cultures.

Isolation using the surface-sterilization method: To isolate the fungus from healthy flowers, leaves, nuts, and husks, a surface sterilization protocol was employed. Tissues were cut into small fragments, surface-sterilized by immersion in 75% ethanol for 30 s, followed by 2% NaOCl for 1 min, and rinsed three times with sterile distilled water. The surface-sterilized fragments were dried on sterile filter paper and placed onto PDA plates. Plates were incubated at 25 °C in the dark and examined daily. Hyphal tips emerging from the tissue edges were transferred to new PDA plates for purification.

### Morphological analyses

Specimens collected from the field were examined to characterize both anamorph and teleomorph. The teleomorph was observed on overwintered leaf litter, while the anamorph was examined on infected fruit husks and in culture. Macroscopic characteristics of the perithecia and pycnidia were observed using a stereomicroscope (Zeiss Discovery V8). High-definition macroscopic photographs were captured to document the appearance of signs on the host substrates. For microscopic examination, hand sections of the fruiting bodies were prepared using a double-edged razor blade under the stereomicroscope. Fungal structures, including asci, ascospores, conidiophores, and conidia, were mounted in distilled water for observation and measurement. Differential interference contrast (DIC) microscopy was performed using an Olympus BX51 microscope (Tokyo, Japan). A total of 50 spores were randomly selected for measurement, with the results presented as maximum and minimum values (in parentheses), along with the range expressed as the mean ± standard deviation.

### Molecular analyses

Genomic DNA was extracted from fresh mycelium grown on PDA at 25 °C for 10 days using the Wizard® Genomic DNA Purification Kit (Promega, Madison, WI, USA) following the manufacturer’s instructions. Three loci were amplified: the internal transcribed spacer regions with the intervening 5.8S nrRNA gene (ITS), the translation elongation factor 1-alpha (*tef*1), and the beta-tubulin (*tub*2). The primers used were ITS1/ITS4 for ITS (White et al. 1990), EF1-728F/EF2 for *tef*1 (Carbone and Kohn 1999), and Bt2a/Bt2b for *tub*2 (Glass and Donaldson 1995). Polymerase chain reaction (PCR) was performed in a 25 μL reaction volume containing 12.5 μL of 2× Taq Master Mix, 1 μL of each primer (10 μM), 1 μL of template DNA, and 9.5 μL of ddH_2_O. The PCR thermal cycling conditions were as follows: an initial denaturation at 95 °C for 5 min, followed by 35 cycles of denaturation at 95 °C for 30 s, annealing at 48 °C (for ITS) or 54 °C (for *tef*1 and *tub*2) for 50 s, and extension at 72 °C for 1 min; and a final extension at 72 °C for 10 min. PCR products were visualized on 1% agarose gel electrophoresis and sequenced by Ruibo Xingke Biotechnology Company Limited (Beijing, China) using the Sanger method.

Forward and reverse sequences were assembled using SeqMan v. 7.1.0. Three species, *Apiognomonia errabunda* AR 2813, *Discula destructiva* CBS 109771 and AR 2817, and *Gnomonia gnomon* CBS 199.53, were selected as the outgroup taxa based on the phylogenetic relationships within *Gnomoniaceae* (Senanayake et al. 2018). The generated sequences were aligned with reference sequences retrieved from GenBank (Suppl. material 1) using the MAFFT v. 7 online server (Katoh and Standley 2013) with the default settings. The alignments were manually adjusted where necessary using MEGA 7 (Kumar et al. 2016). ModelFinder was used to choose the best-fit nucleotide substitution models for each gene: TIM2e+I+G4 for ITS, TIM2+F+R3 for *tef*1, and TVM+F+R4 for *tub*2. Phylogenetic analyses were performed based on the concatenated dataset (ITS-*tef*1-*tub*2) using maximum likelihood (ML) and Bayesian inference (BI). The ML analysis was conducted using the IQ-TREE algorithm via the OFPT software (Zeng et al. 2023). Branch support was evaluated with 1,000 rapid bootstrap replicates. Bayesian inference (BI) was performed using MrBayes v. 3.2.7a (Ronquist et al. 2012). Two independent runs of four simultaneous Markov Chain Monte Carlo (MCMC) chains were executed for 5,000,000 generations, sampling every 1000 generations. The first 25% of sampled trees were discarded as burn-in. The remaining trees were used to calculate the posterior probabilities (PP). Convergence was confirmed when the standard deviation of split frequencies fell below 0.01. Phylogenetic trees were visualized in FigTree v. 1.4.2 (Rambaut 2014), annotated in Adobe Illustrator or Photoshop.

## Results

### Phylogeny

The combined dataset included 131 strains (including the outgroup taxa *Apiognomonia errabunda* AR 2813, *Discula destructiva* CBS 109771 and AR 2817, and *Gnomonia gnomon* CBS 199.53) and comprised 2,334 characters (including gaps) after alignment (ITS: 1–506, *tef*1: 507–1565, *tub*2: 1566–2334). The ML analysis yielded an optimization likelihood value of -26202.72, with the alignment matrix containing 1,356 distinct patterns and 32.49% undetermined characters or gaps. The estimated nucleotide frequencies were as follows: A = 0.221726, C = 0.279192, G = 0.233994, and T = 0.265089. The substitution rates were calculated as AC = 1.272522, AG = 3.345979, AT = 1.411923, CG = 0.889033, CT = 4.333914, and GT = 1.0. The gamma distribution shape parameter (*α*) was estimated at 0.375137. The topology of the ML tree was congruent with that of the Bayesian inference consensus tree.

**Figure 1.**
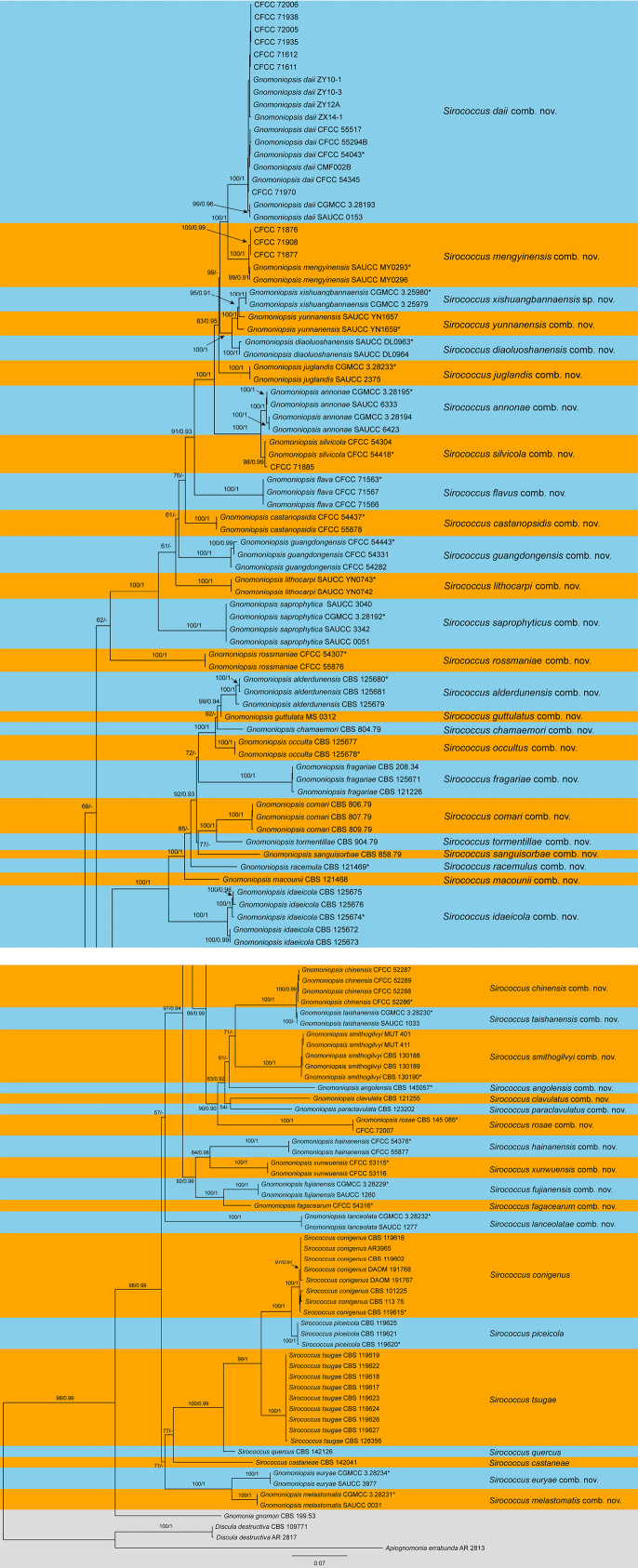
Phylogram of *Sirococcus* resulting from a maximum likelihood analysis based on a multigene matrix of the ITS, *tef*1, and *tub*2 gene loci. Numbers above the branches indicate ML bootstrap support values (left, ML BS ≥ 50%) and Bayesian posterior probabilities (right, BPP ≥ 0.90). *Ex*-type strains are marked with *.

Phylogenetic analyses of the concatenated multi-locus matrix (ITS-*tef*1-*tub*2) revealed a well-supported monophyletic lineage (ML-BS = 98%, BI-PP = 0.99) accommodating all analyzed strains of *Sirococcus* and *Gnomoniopsis* (Fig. 1). Within this major clade, the type species of *Sirococcus*, *S. conigenus* (CBS 119615, *ex*-epitype), and the type species of *Gnomoniopsis*, *Sirococcus chamaemori* (= *Gnomoniopsis chamaemori*, CBS 804.79), were nested together without forming reciprocally monophyletic sister genera. The subclades representing species previously assigned to *Gnomoniopsis* were interspersed with those of *Sirococcussensu stricto*. Consequently, to maintain monophyly, *Gnomoniopsis* is treated as a synonym of *Sirococcus*, and 38 species are recombined in *Sirococcus*, and a new species is proposed herein.

Regarding the newly collected isolates, strains CFCC 72005, CFCC 72006, CFCC 71611, CFCC 71612, and CFCC 71970 obtained from symptomatic nuts and healthy tissues of *Castanea mollissima*, together with CFCC 71935 and CFCC 71938 from symptomatic nuts of *C. henryi*, formed a distinct, fully supported terminal clade (ML-BS = 100%, BI-PP = 1.00) with the *ex*-type strain of *Sirococcus daii* (CFCC 54043). Isolates CFCC 71876, CFCC 71877, and CFCC 71908 from healthy nuts of *C. mollissima* clustered in a fully supported clade with the *ex*-type strain of *S. mengyinensis* (SAUCC MY0293). Additionally, the isolate CFCC 72007 from a healthy leaf of *Rosa chinensis* clustered with the *ex*-type strain of *S. rosae* (CBS 145085), while isolate CFCC 71885 from a rotten nut of *Castanopsis carlesii* formed a clade with the *ex*-type strain of *S. silvicola* (CFCC 54418).

### Taxonomy

#### Sirococcus

Taxon classificationAnimaliaDiaporthalesGnomoniaceae

Preuss, Linnaea 26: 716 (1855)

A808D757-5BD9-5C25-B7F3-5FCD847C96F1

9927

##### Synonym.

*Gnomoniopsis* Berl., Icon. fung. (Abellini) 1(3): 93 (1893).

##### Description.

***Ascomata*** solitary, lacking stroma, or in groups, aggregated in minimal pseudostroma, black, globose to subglobose, concave from base when dry. *Neck* central to lateral, straight or slightly curved to curved, short to long, sometimes almost absent, in cross section circular to oval or flattened. *Asci* 8-spored, unitunicate, ellipsoid, ovoid, obovoid, to fusiform, with conspicuous apical ring. *Ascospores* uniseriate, biseriate, or irregularly multiseriate, fusiform, obovoid or pyriform, one-septate, or aseptate, median to submedian, constricted or not at septum, hyaline, lacking appendages. ***Conidiomata*** pycnidial, eustromatic, glabrous, erumpent without a clypeus, globose to flattened, black, coriaceous. *Peridium* comprising multilayered, brown cells of textura angularis. *Conidiophores* simple or branched, septate. *Conidiogenous cells* monophialidic. *Conidia* hyaline, median-septate, fusiform.

##### Type species.

*Sirococcus conigenus* (Pers.) P.F. Cannon & Minter, Taxon 32(4): 577 (1983).

##### Basionym.

*Hysterium conigenum* Pers., Observ. mycol. (Lipsiae) 1: 30 (1796).

#### Sirococcus alderdunensis

Taxon classificationAnimaliaDiaporthalesGnomoniaceae

(D.M. Walker) Ning Jiang comb. nov.

543A8327-FF40-546E-9B82-57CBCD9788F0

862090

##### Basionym.

*Gnomoniopsis alderdunensis* D.M. Walker [as *‘alderdunense’*], Mycologia 102(6): 1487 (2010).

#### Sirococcus angolensis

Taxon classificationAnimaliaDiaporthalesGnomoniaceae

(Crous) Ning Jiang comb. nov.

B0891014-BBD2-5799-A823-2DCC7D1FB8F0

862091

##### Basionym.

*Gnomoniopsis angolensis* Crous, Persoonia 41: 265 (2018).

#### Sirococcus annonae

Taxon classificationAnimaliaDiaporthalesGnomoniaceae

(Zhao X. Zhang & X.G. Zhang) Ning Jiang comb. nov.

448DA1E6-468E-5510-A5C4-878CED04AA25

862092

##### Basionym.

*Gnomoniopsis annonae* Zhao X. Zhang & X.G. Zhang, Fungal Diversity 132: 74 (2025).

#### Sirococcus castanopsidis

Taxon classificationAnimaliaDiaporthalesGnomoniaceae

(Ning Jiang) Ning Jiang comb. nov.

8238C8F3-E3D1-5FF1-9970-903AE58BA756

862093

##### Basionym.

*Gnomoniopsis castanopsidis* Ning Jiang, J. Fungi 7(10, no. 792): 8 (2021).

#### Sirococcus chamaemori

Taxon classificationAnimaliaDiaporthalesGnomoniaceae

(Fr.) Ning Jiang comb. nov.

76BC1A0F-E18A-56B7-9B35-A207B5208C32

862094

≡ Gnomoniopsis chamaemori (Fr.) Berl., Icon. fung. (Abellini) 1(3): 93 (1893).

##### Basionym.

*Sphaeria chamaemori* Fr., Systema Mycologicum 2(2): 519 (1823).

#### Sirococcus chinensis

Taxon classificationAnimaliaDiaporthalesGnomoniaceae

(C.M. Tian & Ning Jiang) Ning Jiang comb. nov.

C100D160-EABF-5001-ADE2-0342AF78F1FB

862095

##### Basionym.

*Gnomoniopsis chinensis* C.M. Tian & Ning Jiang, MycoKeys 67: 24 (2020).

#### Sirococcus clavulatus

Taxon classificationAnimaliaDiaporthalesGnomoniaceae

(Ellis) Ning Jiang comb. nov.

999244EF-C6DC-5646-B190-71464C208B42

862096

≡ Gnomoniopsis clavulata (Ellis) Sogonov, Stud. Mycol. 62: 44 (2008).

##### Basionym.

*Gnomonia clavulata* Ellis, Am. Nat. 17: 318 (1883).

#### Sirococcus comari

Taxon classificationAnimaliaDiaporthalesGnomoniaceae

(P. Karst.) Ning Jiang comb. nov.

C53031B2-D4F2-527F-B366-52A7DF05E1B6

862097

≡ Gnomoniopsis comari (P. Karst.) Sogonov, Mycologia 102(6): 1487 (2010).

##### Basionym.

*Gnomonia comari* P. Karst., Fung. Fenn. Exsicc., Cent. 9(nos 800–900): no. 869 (1869).

#### Sirococcus daii

Taxon classificationAnimaliaDiaporthalesGnomoniaceae

(C.M. Tian & Ning Jiang) Ning Jiang comb. nov.

4BE6188F-95BA-55CB-990B-211015BE949E

862098

Figs 2, 3

##### Basionym.

*Gnomoniopsis daii* C.M. Tian & Ning Jiang, Forests 10(11/1016): 6 (2019).

##### Description.

***Perithecia*** hypophyllous or less commonly epiphyllous, serried, immersed, subepidermal, black, oblate spheroidal, 110–180 μm diam. *Necks* absent. *Asci* hyaline, with chitinoid, refractive ring, fusiform or obclavate, 58.5–81 × 6.5–9.5 μm, 8-spored. *Ascospores* biseriate, fusiform, straight to slightly curved, hyaline, aseptate, guttulate, (15.5–)18–23(–24.5) × (4–)4.5–5(–5.5) μm (mean = 20.6 × 4.7, n = 50), l/w = 3.7–5.2. ***Conidiomata*** pycnidial, eustromatic, glabrous, flask-shaped, base black, neck yellow to orange, 130–200 μm diam. *Peridium* comprising multilayered, brown cells of textura angularis. *Conidiophores* indistinct, often reduced to conidiogenous cells. *Conidiogenous cells* enteroblastic, phialidic, subcylindrical to cylindrical, 7–11.5 × 1.5–3.5 μm. *Conidia* aseptate, hyaline, smooth, guttulate, cylindrical, straight, base truncate, (5.5–)6–6.5 × 2–2.5 μm (mean = 6.1 × 2.2, n = 50), l/w = 2.5–3.

**Figure 2.**
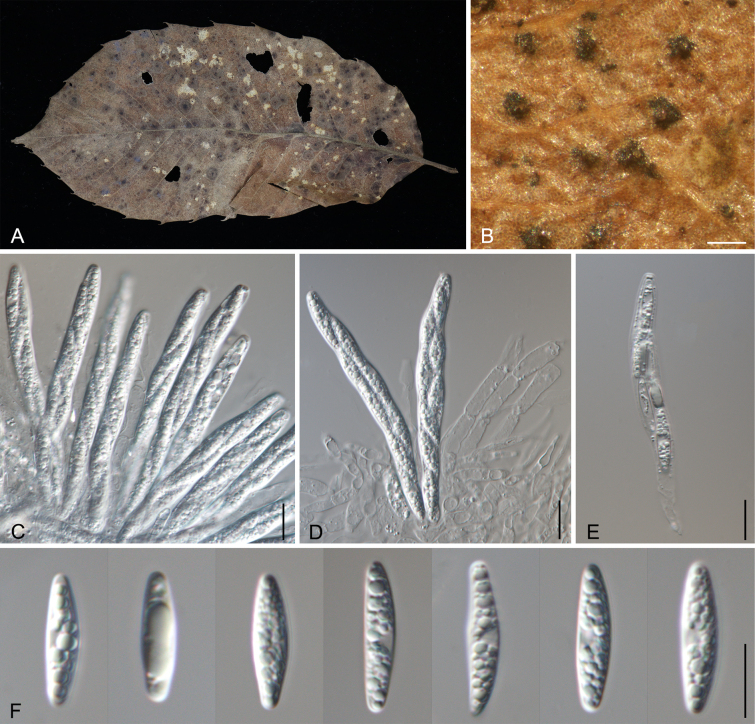
Teleomorph of *Sirococcus daii*. **A, B**. Habit of ascomata on a leaf. **C–E**. Asci and ascospores. **F**. Ascospores. Scale bars: 200 μm (**B**); 20 μm (**C–E**); 10 μm (**F**).

##### Materials examined.

China • Shaanxi Province, Ankang City, chestnut plantation, on the fallen husks of *Castanea mollissima*, 22 March 2025, Ning Jiang (culture CFCC 71611 from a conidium); Fujian Province, Nanping City, chestnut plantation, on the fallen leaves of *C. mollissima*, 8 December 2025, Ning Jiang (culture CFCC 71970 from an ascospore); Fujian Province, Jianou City, chinquapin plantation, on the healthy nut of *C. henryi*, 12 October 2025, Ning Jiang (culture CFCC 71935 from a healthy chinquapin nut); Fujian Province, Jianou City, chinquapin plantation, on the rotten nut of *C. henryi*, 12 October 2025, Ning Jiang (culture CFCC 71938 from a rotten chinquapin nut); Beijing City, Huairou District, chestnut plantation, on the female flowers of *C. mollissima*, 27 May 2024, Ning Jiang (culture CFCC 72005 from a healthy chestnut flower); Beijing City, Huairou District, chestnut plantation, on a healthy leaf of *C. mollissima*, 27 May 2024, Ning Jiang (culture CFCC 72006 from a healthy chestnut leaf); Beijing City, Huairou District, chestnut plantation, on a developing nut of *C. mollissima*, 12 July 2024, Ning Jiang (culture CFCC 71612 from a developing nut).

**Figure 3.**
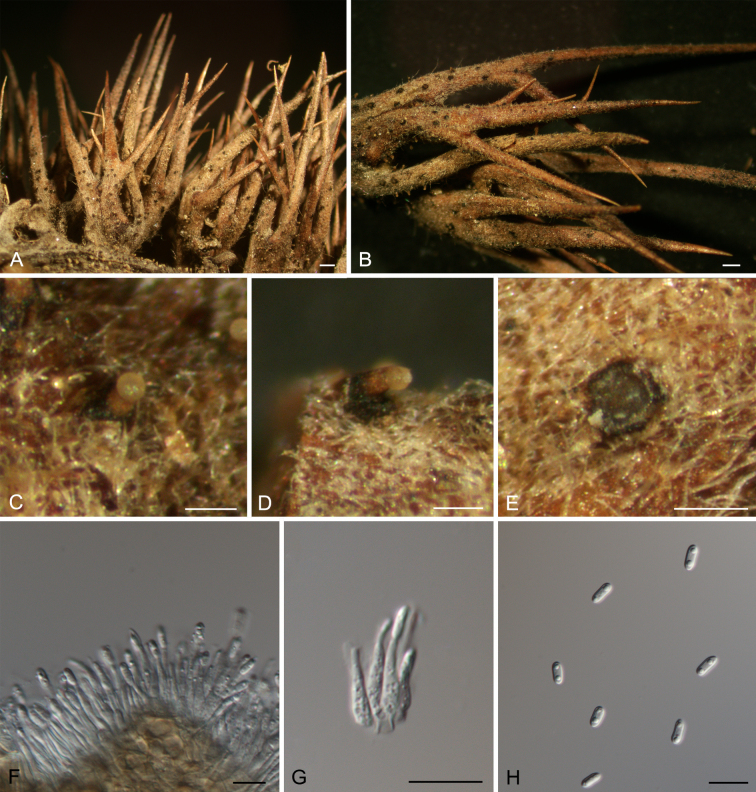
Anamorph of *Sirococcus daii*. **A, B**. Habit of conidiomata on the husks. **C, D**. Conidiomata. **E**. Transverse section through conidioma. **F, G**. Conidiogenous cells with attached conidia. **H**. Conidia. Scale bars: 300 μm (**A–E**); 10 μm (**F–H**).

##### Notes.

*Gnomoniopsis daii* was proposed as the causal agent of Chinese chestnut nut rot (Jiang and Tian 2019), and later its natural anamorph was reported from leaf spots of *Castanea mollissima* (Jiang et al. 2021a). In this study, we first discovered its teleomorph on fallen leaves and its anamorph on the husks. Additionally, this fungus is combined into *Sirococcus* as *S. daii*.

#### Sirococcus diaoluoshanensis

Taxon classificationAnimaliaDiaporthalesGnomoniaceae

(Shi Wang, Zhao X. Zhang, X.Y. Liu & X.G. Zhang) Ning Jiang comb. nov.

E8AEE07C-3E10-5A94-9F37-FAF723716696

862099

##### Basionym.

*Gnomoniopsis diaoluoshanensis* Shi Wang, Zhao X. Zhang, X.Y. Liu & X.G. Zhang, J. Fungi 8(8, no. 770): 5 (2022).

#### Sirococcus euryae

Taxon classificationAnimaliaDiaporthalesGnomoniaceae

(Zhao X. Zhang & X.G. Zhang) Ning Jiang comb. nov.

85AEAC5E-0ED6-556D-923F-E4ACF43B01E2

862100

##### Basionym.

*Gnomoniopsis euryae* Zhao X. Zhang & X.G. Zhang, Fungal Diversity 132: 76 (2025).

#### Sirococcus fagacearum

Taxon classificationAnimaliaDiaporthalesGnomoniaceae

(Ning Jiang) Ning Jiang comb. nov.

C89F1556-3139-521B-B026-74BB49A6BE6D

862101

##### Basionym.

*Gnomoniopsis fagacearum* Ning Jiang, J. Fungi 7(10, no. 792): 10 (2021).

#### Sirococcus flavus

Taxon classificationAnimaliaDiaporthalesGnomoniaceae

(Ning Jiang) Ning Jiang comb. nov.

D2C8DB6B-9AB8-56DB-BCFE-8634E880805D

862102

##### Basionym.

*Gnomoniopsis flava* Ning Jiang, Forests 16(4, no. 627): 7 (2025).

#### Sirococcus fragariae

Taxon classificationAnimaliaDiaporthalesGnomoniaceae

(Laib.) Ning Jiang comb. nov.

6C27EBE2-753D-5814-A89D-012FB7CD2E93

862103

≡ Gnomoniopsis fragariae (Laib.) Udayanga & Castl., IMA Fungus 12(no. 15): 12 (2021).

##### Basionym.

*Zythia fragariae* Laib., Arb. K. biol. Anst. f. Land-u-Forstwirt. 6: 79 (1908).

#### Sirococcus fujianensis

Taxon classificationAnimaliaDiaporthalesGnomoniaceae

(Zhao X. Zhang & X.G. Zhang) Ning Jiang comb. nov.

CF78BFC8-D059-5081-A942-00261A6804AA

862104

##### Basionym.

*Gnomoniopsis fujianensis* Zhao X. Zhang & X.G. Zhang, Fungal Diversity 132: 77 (2025).

#### Sirococcus guangdongensis

Taxon classificationAnimaliaDiaporthalesGnomoniaceae

(Ning Jiang) Ning Jiang comb. nov.

4307F829-3E44-5DB0-9BB6-86F0349F07E9

862105

##### Basionym.

*Gnomoniopsis guangdongensis* Ning Jiang, J. Fungi 7(10, no. 792): 11 (2021).

#### Sirococcus guttulatus

Taxon classificationAnimaliaDiaporthalesGnomoniaceae

(Starbäck) Ning Jiang comb. nov.

28C52516-24B0-5641-9ADB-19669FDCCA05

862106

≡ Gnomoniopsis guttulata (Starbäck) D.M. Walker, Mycologia 102(6): 1490 (2010).

##### Basionym.

*Gnomoniella guttulata* Starbäck, Bih. K. svenska Vetensk. Akad. Handl., Afd. 3 15(no. 2): 10 (1889).

#### Sirococcus hainanensis

Taxon classificationAnimaliaDiaporthalesGnomoniaceae

(Ning Jiang) Ning Jiang comb. nov.

8E457B4E-353D-5C19-ACA2-53C951321D53

862107

##### Basionym.

*Gnomoniopsis hainanensis* Ning Jiang, J. Fungi 7(10, no. 792): 12 (2021).

#### Sirococcus idaeicola

Taxon classificationAnimaliaDiaporthalesGnomoniaceae

(P. Karst.) Ning Jiang comb. nov.

B4304FEC-03F7-5C3E-9C84-3DC9EEABF280

862108

≡ Gnomoniopsis idaeicola (P. Karst.) D.M. Walker, Mycologia 102(6): 1490 (2010).

##### Basionym.

*Calosphaeria idaeicola* P. Karst., Fung. Fenn. Exsicc., Cent. 9(nos 800–900): no. 856 (1869).

#### Sirococcus juglandis

Taxon classificationAnimaliaDiaporthalesGnomoniaceae

(Zhao X. Zhang & X.G. Zhang) Ning Jiang comb. nov.

C91339C1-0F92-5A4F-BD66-962A7641FB29

862109

##### Basionym.

*Gnomoniopsis juglandis* Zhao X. Zhang & X.G. Zhang, Fungal Diversity 132: 78 (2025).

#### Sirococcus lanceolatae

Taxon classificationAnimaliaDiaporthalesGnomoniaceae

(Zhao X. Zhang & X.G. Zhang) Ning Jiang comb. nov.

9D15F19D-D666-5F61-A3F3-D43BA44B1933

862110

##### Basionym.

*Gnomoniopsis lanceolatae* Zhao X. Zhang & X.G. Zhang, Fungal Diversity 132: 79 (2025) [as ‘*lanceolata’*].

#### Sirococcus lithocarpi

Taxon classificationAnimaliaDiaporthalesGnomoniaceae

(Shi Wang, Zhao X. Zhang, X.Y. Liu & X.G. Zhang) Ning Jiang comb. nov.

86110D5E-B6C0-5A9D-80F6-72D594D378B6

862111

##### Basionym.

*Gnomoniopsis lithocarpi* Shi Wang, Zhao X. Zhang, X.Y. Liu & X.G. Zhang, J. Fungi 8(8, no. 770): 7 (2022).

#### Sirococcus macounii

Taxon classificationAnimaliaDiaporthalesGnomoniaceae

(Dearn.) Ning Jiang comb. nov.

90B3D77B-5D65-5607-A260-9E449EC35A37

862112

≡ Gnomoniopsis macounii (Dearn.) Sogonov, Stud. Mycol. 62: 48 (2008).

##### Basionym.

*Diaporthe macounii* Dearn., Mycologia 8(2): 100 (1916).

#### Sirococcus melastomatis

Taxon classificationAnimaliaDiaporthalesGnomoniaceae

(Zhao X. Zhang & X.G. Zhang) Ning Jiang comb. nov.

FE4282C9-9031-527B-A33B-D90456006647

862113

##### Basionym.

*Gnomoniopsis melastomatis* Zhao X. Zhang & X.G. Zhang, Fungal Diversity 132: 80 (2025).

#### Sirococcus mengyinensis

Taxon classificationAnimaliaDiaporthalesGnomoniaceae

(Shi Wang, Zhao X. Zhang, X.Y. Liu & X.G. Zhang) Ning Jiang comb. nov.

F3FF6D3C-5D4C-5BFA-8063-BD3EA9D0C0BB

862114

##### Basionym.

*Gnomoniopsis mengyinensis* Shi Wang, Zhao X. Zhang, X.Y. Liu & X.G. Zhang, J. Fungi 8(8, no. 770): 9 (2022).

##### Materials examined.

China • Guizhou Province, Anshun City, Puding County, chestnut plantation, on healthy nuts of *Castanea mollissima*, 15 October 2025, Zixuan Li (tissue-isolated cultures CFCC 71876, CFCC 71877); Jiangxi Province, Shangrao City, Yushan County, chestnut plantation, on healthy nut of *C. mollissima*, 5 November 2025, Zixuan Li (tissue-isolated culture CFCC 71908).

##### Notes.

Three new isolates are identified as *Gnomoniopsis mengyinensis*, with sequences perfectly matching those of Wang et al. (2022). Here, this fungus is transferred to *Sirococcus* as *S. mengyinensis*.

#### Sirococcus occultus

Taxon classificationAnimaliaDiaporthalesGnomoniaceae

(Kirschst.) Ning Jiang comb. nov.

BB0E18B2-A878-56C9-9380-09221A488858

862115

≡ Gnomoniopsis occulta (Kirschst.) D.M. Walker, Mycologia 102(6): 1492 (2010).

##### Basionym.

*Gnomonia occulta* Kirschst., Verh. bot. Ver. Prov. Brandenb. 48: 58 (1906).

#### Sirococcus paraclavulatus

Taxon classificationAnimaliaDiaporthalesGnomoniaceae

(Sogonov) Ning Jiang comb. nov.

03A1CD69-6FB1-5FC7-89D6-E392BC0BBB19

862116

##### Basionym.

*Gnomoniopsis paraclavulata* Sogonov, Stud. Mycol. 62: 44 (2008).

#### Sirococcus racemulus

Taxon classificationAnimaliaDiaporthalesGnomoniaceae

(Cooke & Peck) Ning Jiang comb. nov.

BDDC3ADD-FB8A-598E-8CB0-5BADAC335F9E

862117

≡ Gnomoniopsis racemula (Cooke & Peck) Sogonov, Stud. Mycol. 62: 48 (2008).

##### Basionym.

*Sphaeria racemula* Cooke & Peck, in Peck, Ann. Rep. N.Y. St. Mus. nat. Hist. 29: 65 (1878).

#### Sirococcus rosae

Taxon classificationAnimaliaDiaporthalesGnomoniaceae

(Crous) Ning Jiang comb. nov.

A9E0B20C-9907-55E3-8F84-72F3558B5349

862118

##### Basionym.

*Gnomoniopsis rosae* Crous, Persoonia 41: 305 (2018).

##### Materials examined.

China • Beijing City, Changping District, on a healthy leaf of *Rosa chinensis*, 8 July 2024, Ning Jiang (tissue-isolated culture CFCC 72007).

##### Notes.

*Gnomoniopsis rosae* was proposed by Crous et al. (2018) and discovered in China on the same host genus, *Rosa* (Li et al. 2023). Here, a new isolate CFCC 72007 is identified as this fungus based on molecular phylogeny (Fig. 1). In addition, this species is transferred to *Sirococcus* as *S. rosae*.

#### Sirococcus rossmaniae

Taxon classificationAnimaliaDiaporthalesGnomoniaceae

(Ning Jiang) Ning Jiang comb. nov.

62CD6520-137E-571E-9D11-6B404C7F91EC

862119

##### Basionym.

*Gnomoniopsis rossmaniae* Ning Jiang, J. Fungi 7(10, no. 792): 13 (2021).

#### Sirococcus sanguisorbae

Taxon classificationAnimaliaDiaporthalesGnomoniaceae

(Rehm) Ning Jiang comb. et stat. nov.

99EDAF17-2189-59D8-8D13-C5835BB22192

862120

≡ Gnomoniopsis sanguisorbae (Rehm) D.M. Walker, Mycologia 102(6): 1494 (2010).

##### Basionym.

*Gnomonia tithymalina* var. *sanguisorbae* Rehm, Annls mycol. 3(3): 229 (1905).

#### Sirococcus saprophyticus

Taxon classificationAnimaliaDiaporthalesGnomoniaceae

(Zhao X. Zhang & X.G. Zhang) Ning Jiang comb. nov.

A00D70C4-B7F3-5540-A4BD-480F1DD1A572

862121

##### Basionym.

*Gnomoniopsis saprophytica* Zhao X. Zhang & X.G. Zhang, Fungal Diversity 132: 81 (2025).

#### Sirococcus silvicola

Taxon classificationAnimaliaDiaporthalesGnomoniaceae

(Ning Jiang) Ning Jiang comb. nov.

82A6499A-1173-580E-A5A4-4E11FBE753F5

862122

##### Basionym.

*Gnomoniopsis silvicola* Ning Jiang, Fungi 7(10, no. 792): 14 (2021).

##### Materials examined.

China • Jiangxi Province, Ganzhou City, Ruijin City, on healthy nut of *Castanopsis carlesii*, 15 October 2025, Yan Liu (tissue-isolated culture CFCC 71885).

##### Notes.

*Gnomoniopsis silvicola* was established from leaf spots of *Castanopsis hystrix* and *Quercus serrata* in China (Jiang et al. 2021b). Here, we identified an isolate from the healthy nut of *Castanopsis carlesii* as this species and combined this fungus in the genus *Sirococcus*. Hence, *C. carlesii* is a new host for *Sirococcus silvicola*.

#### Sirococcus smithogilvyi

Taxon classificationAnimaliaDiaporthalesGnomoniaceae

(L.A. Shuttlew., E.C.Y. Liew & D.I. Guest) Ning Jiang comb. nov.

349325B7-26FE-542D-94FF-3501B49FA169

862123

= *Gnomoniopsis castaneae* Tamietti [as *‘castanea’*], Journal of Plant Pathology 94(2): 412 (2012).

##### Basionym.

*Gnomoniopsis smithogilvyi* L.A. Shuttlew., E.C.Y. Liew & D.I. Guest, Persoonia 28: 143 (2012).

#### Sirococcus taishanensis

Taxon classificationAnimaliaDiaporthalesGnomoniaceae

(Zhao X. Zhang & X.G. Zhang) Ning Jiang comb. nov.

26AD24F0-A380-519B-A086-205DA5431CA4

862124

##### Basionym.

*Gnomoniopsis taishanensis* Zhao X. Zhang & X.G. Zhang, Fungal Diversity 132: 82 (2025).

#### Sirococcus tormentillae

Taxon classificationAnimaliaDiaporthalesGnomoniaceae

(Lind) Ning Jiang comb. nov.

D51655B1-9954-5F9D-8276-3B2C833B7DA5

862125

≡ Gnomoniopsis tormentillae (Lind) Sogonov, Stud. Mycol. 62: 48 (2008).

##### Basionym.

*Gnomoniella tormentillae* Lind, Bot. Tidsskr. 41(3): 217 (1928).

#### Sirococcus xishuangbannaensis

Taxon classificationAnimaliaDiaporthalesGnomoniaceae

Zhao X. Zhang & X.G. Zhang ex Ning Jiang sp. nov.

6C4921E9-2969-5473-B4D3-295B218EC44D

862669

≡ Gnomoniopsis xishuangbannaensis Zhao X. Zhang & X.G. Zhang, in Zhang, Shang, Liu, Li, Yin, Liu, Tao, Jiang, Wang, Zhang, Dong, Yun, Xia, Wang, Li, Luo, Liu & Zhang, Fungal Diversity 132: 83 (2025), nom. inval., Art. 40.7 (Shenzhen).

##### Holotype.

HSAUP 1635.

For an effectively published description, see Zhang et al. (2025).

##### Notes.

*Gnomoniopsis xishuangbannaensis* was originally introduced by Zhang et al. (2025) with a full description and illustration. However, the authors designated two specimens (HSAUP 1635 and HSAUP 1642) as holotypes, which contravenes Art. 40.7 of the ICN (Shenzhen Code), rendering the name invalidly published. In this study, we validate the name by selecting a single specimen (the first accession code), HSAUP 1635, as the holotype.

#### Sirococcus xunwuensis

Taxon classificationAnimaliaDiaporthalesGnomoniaceae

(C.M. Tian & Qin Yang) Ning Jiang comb. nov.

D0C1905A-9767-5DC4-9CCE-ABDFF84E5449

862127

##### Basionym.

*Gnomoniopsis xunwuensis* C.M. Tian & Qin Yang, MycoKeys 69: 82 (2020).

#### Sirococcus yunnanensis

Taxon classificationAnimaliaDiaporthalesGnomoniaceae

(Shi Wang, Zhao X. Zhang, X.Y. Liu & X.G. Zhang) Ning Jiang comb. nov.

85511252-F9F9-59F8-8D45-6C26AF7E646F

862128

##### Basionym.

*Gnomoniopsis yunnanensis* Shi Wang, Zhao X. Zhang, X.Y. Liu & X.G. Zhang, J. Fungi 8(8, no. 770): 10 (2022).

### Life cycle of *Sirococcus daii*

**Figure 4.**
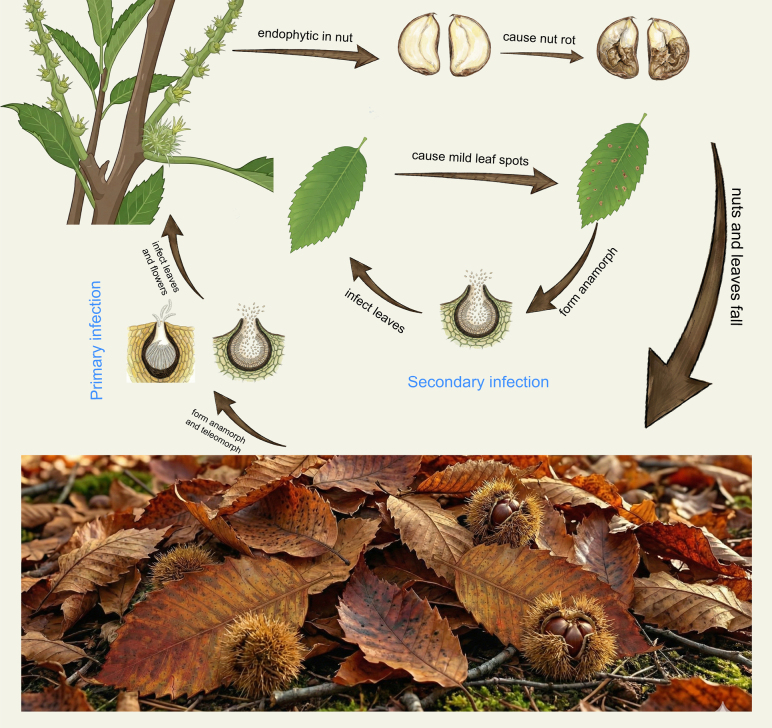
Hypothetical life cycle of *Sirococcus daii* on *Castanea mollissima*. The cycle initiates with primary infection in spring, where ascospores and conidia released from overwintered leaf litter and husks infect young leaves and female flowers. The fungus maintains an endophytic phase in developing nuts and causes mild leaf spots on leaves. Conidia produced on leaf spots serve as inoculum for secondary infection. As nuts mature, the fungus causes severe nut rot. In autumn, infected nuts and leaves fall to the ground, where the fungus survives saprotrophically, overwinters, and forms perithecia and pycnidia, completing the cycle.

Based on the field observations and isolation results from diverse host tissues, the life cycle of *Sirococcus daii* on *Castanea mollissima* was reconstructed (Fig. 4). The fungus exhibits a hemibiotrophic lifestyle, alternating between an endophytic/latent phase in living tissues and a saprotrophic phase on decaying debris.

Overwintering and Primary Infection: The pathogen overwinters as both perithecia and pycnidia on fallen leaf litter and pycnidia on decaying fruit husks. In the following spring, under warm and humid conditions, ascospores released from perithecia and conidia released from pycnidia serve as the primary inoculum. These spores are dispersed by wind or rain splash to colonize the emerging tender tissues of the host, including young leaves and female flowers.

Latent Phase and Symptom Development: Following the primary infection, *S. daii* establishes a latent or endophytic infection within the host tissues. In leaves, the fungus may cause mild symptoms appearing as small necrotic leaf spots. On these lesions, pycnidia are produced, releasing conidia that serve as secondary inoculum to infect adjacent leaves, driving a polycyclic infection during the growing season. Crucially, the fungus colonizes the female flowers and developing nuts without initially causing visible symptoms. It persists endophytically within the developing nut kernels throughout the summer. As the nuts approach maturity, the fungus switches to a necrotrophic phase, leading to the typical nut rot symptoms, characterized by the browning and necrosis of the kernel.

Cycle Completion: In late autumn, infected leaves, husks, and rotten nuts fall to the ground. The fungus continues to survive saprotrophically on this debris. During the winter months, *S. daii* develops perithecia and pycnidia on the overwintering substrate, completing the disease cycle and preparing the inoculum for the next season.

## Discussion

The generic boundaries within *Gnomoniaceae* have long been a subject of debate, primarily due to the historical separation of taxa based on morph states and host associations (Shuttleworth et al. 2016; Shuttleworth and Guest 2017; Senanayake et al. 2018; Jiang and Tian 2019; Jiang et al. 2020, 2021b; Yang et al. 2020). Our study represents the most comprehensive phylogenetic re-evaluation of the *Sirococcus/Gnomoniopsis* complex to date. By incorporating the type species of both genera (*S. conigenus* and *G. chamaemori*) into a multi-locus phylogeny (ITS, *tef*1, *tub*2), we resolved a robust monophyletic lineage that accommodates species previously treated as distinct genera.

The decision to synonymize *Gnomoniopsis* under *Sirococcus* is grounded in both phylogenetic evidence and nomenclatural priority. Phylogenetic analyses (Fig. 1) clearly demonstrate that maintaining both genera as distinct entities renders *Gnomoniopsis* paraphyletic, as the strongly supported monophyletic clade of *Sirococcussensu stricto* (i.e., the conifer-inhabiting species) is nested among taxa previously assigned to *Gnomoniopsis*. Resolving this paraphyly would necessitate the fragmentation of the broader clade into multiple, morphologically indistinguishable genera (Rossman et al. 2008; Walker et al. 2010; Visentin et al. 2012; Crous et al. 2013; Jiang et al. 2021b; Wang et al. 2022; Zhang et al. 2025). Such splitting would result in taxonomic instability and practical confusion. Under the “One Fungus, One Name” principle and Article 11 of the Shenzhen Code (ICN), *Sirococcus*Preuss (1855) holds clear priority over *Gnomoniopsis* Berl. (1893).

This synonymy has profound implications for the generic concept of *Sirococcus*. Historically, *Sirococcus* was viewed as a genus of conifer pathogens causing shoot blights (Bahnweg et al. 2000; Smith et al. 2003; Rossman et al. 2008). However, our proposed 38 new combinations and a new species expand the genus to include pathogens and endophytes of a wide range of broad-leaved hosts, particularly *Fagaceae* (chestnuts, oaks) and *Rosaceae* (Walker et al. 2010; Jiang et al. 2020, 2021b; Wang et al. 2022; Li et al. 2025). This expansion reflects a more natural evolutionary history within a single generic lineage. Specifically, the phylogenetic topology suggests that the ancestor of this lineage was likely associated with diverse angiosperms, followed by a single host-jump event to gymnosperms that gave rise to the strongly supported monophyletic clade of *Sirococcussensu stricto* (Fig. 1). The unification resolves the long-standing artificial dichotomy between the “conifer-associated *Sirococcus*” and the “hardwood-associated *Gnomoniopsis*,” providing a stable framework for future taxonomic and pathological studies.

The synonymy proposed herein is not solely molecularly driven but is also morphologically congruent. Historically, the distinction between these genera was largely artifactual: *Sirococcus* was defined by its coelomycetous anamorph, while *Gnomoniopsis* was defined by its perithecial teleomorph (Rossman et al. 2008; Sogonov et al. 2008). Our study, along with recent literature, confirms that this distinction is biologically unfounded (Rossman et al. 2008; Sogonov et al. 2008; Jiang and Tian 2019; Wang et al. 2022; Zhang et al. 2025).

We observed that species within the expanded *Sirococcus* share a highly conserved asexual morphology: pycnidial conidiomata, phialidic conidiogenous cells producing hyaline, fusiform to cylindrical conidia (Rossman et al. 2008; Senanayake et al. 2018). This specific suite of characters is a reliable synapomorphy for the genus *Sirococcus*. For instance, the anamorph of the type species *G. chamaemori* and the chestnut pathogen *G. smithogilvyi* are morphologically indistinguishable from the classic *Sirococcus* anamorph (Sogonov et al. 2008; Shuttleworth et al. 2016).

Critically, a teleomorph has never been reported for any species within *Sirococcussensu stricto*. This complete absence likely reflects a lack of targeted sampling of overwintered debris during the appropriate season, rather than an inherent inability to reproduce sexually (Rossman et al. 2008). Our discovery of the teleomorph of *S. daii* supports the hypothesis that many “anamorphic” *Sirococcus* species possess cryptic sexual morphs that develop on overwintered debris. Therefore, the unification of these genera successfully integrates the pleomorphic life stages into a single holistic generic concept.

A significant contribution of this study is the complete characterization of *Sirococcus daii* (formerly *Gnomoniopsis daii*). Since its initial report as the causal agent of chestnut nut rot in China, this pathogen has been known only from its anamorph (Jiang and Tian 2019; Li et al. 2025). The lack of teleomorph information hampered its accurate taxonomic placement and biological understanding. We provide the first description of the teleomorph of *S. daii*, found on overwintered leaf litter. The perithecia of *S. daii* exhibit an atypical feature of this genus: the lack of elongated perithecial necks (Sogonov et al. 2008; Walker et al. 2010). Phylogenetically, *S. daii* forms a distinct lineage separate from *S. mengyinensis*, confirming its status as a distinct species rather than a host-specific variant.

The elucidation of the teleomorph is crucial for understanding the reproductive strategy of the species. The presence of both teleomorph and anamorph on overwintered debris suggests that *S. daii* is homothallic or that compatible mating types are widespread in the field. This capability for sexual recombination may contribute to the pathogen’s genetic diversity and adaptability, posing potential challenges for long-term resistance breeding in Chinese chestnut.

The reconstruction of the *S. daii* life cycle (Fig. 4) provides critical insights into the epidemiology of chestnut nut rot. Previously, control measures primarily focused on the nut-growth stage when symptoms became visible (Jiang and Tian 2019). However, our study reveals that *S. daii* behaves as a hemibiotroph with a significant latent/endophytic phase.

Our isolation of *S. daii* from healthy female flowers and developing nuts indicates that infection occurs very early in the season, likely during flowering, but remains asymptomatic until nut maturity. This “flower-to-nut” infection pathway parallels that of *S. smithogilvyi* (Shuttleworth and Guest 2017). Furthermore, the identification of overwintered litter as the primary source of inoculum (producing both ascospores and conidia) highlights a critical vulnerability in the disease cycle.

These epidemiological insights compel a fundamental restructuring of disease management strategies for *S. daii*. Given that the pathogen overwinters on forest debris, management protocols must prioritize the rigorous elimination of fallen leaves and burrs to reduce the primary inoculum pressure. Furthermore, the discovery of the “flower-to-nut” latent infection pathway explains why fungicide applications during the visible rot stage are largely futile; the pathogen is already established within the kernel. Consequently, chemical control must shift from curative to prophylactic, strictly targeting the flowering and early fruit-setting stages to block initial colonization. Finally, the confirmation of *S. daii* as a foliar endophyte implies that the canopy itself acts as a persistent reservoir for secondary inoculum. This suggests that in addition to sanitation, canopy management to improve aeration and the use of systemic fungicides may be necessary to interrupt the polycyclic infection chain (Shuttleworth and Guest 2017).

In conclusion, this study resolves a major taxonomic conflict in *Gnomoniaceae* by synonymizing *Gnomoniopsis* under *Sirococcus* and proposing 38 new combinations and a new species. This revision establishes *Sirococcus* as a large, biologically diverse genus associated with both gymnosperms and angiosperms. Furthermore, by elucidating the full life cycle of *S. daii* and discovering its teleomorph, we provide the biological foundation necessary for developing effective management strategies against this emerging threat to the chestnut industry.

## Supplementary Material

XML Treatment for Sirococcus

XML Treatment for Sirococcus alderdunensis

XML Treatment for Sirococcus angolensis

XML Treatment for Sirococcus annonae

XML Treatment for Sirococcus castanopsidis

XML Treatment for Sirococcus chamaemori

XML Treatment for Sirococcus chinensis

XML Treatment for Sirococcus clavulatus

XML Treatment for Sirococcus comari

XML Treatment for Sirococcus daii

XML Treatment for Sirococcus diaoluoshanensis

XML Treatment for Sirococcus euryae

XML Treatment for Sirococcus fagacearum

XML Treatment for Sirococcus flavus

XML Treatment for Sirococcus fragariae

XML Treatment for Sirococcus fujianensis

XML Treatment for Sirococcus guangdongensis

XML Treatment for Sirococcus guttulatus

XML Treatment for Sirococcus hainanensis

XML Treatment for Sirococcus idaeicola

XML Treatment for Sirococcus juglandis

XML Treatment for Sirococcus lanceolatae

XML Treatment for Sirococcus lithocarpi

XML Treatment for Sirococcus macounii

XML Treatment for Sirococcus melastomatis

XML Treatment for Sirococcus mengyinensis

XML Treatment for Sirococcus occultus

XML Treatment for Sirococcus paraclavulatus

XML Treatment for Sirococcus racemulus

XML Treatment for Sirococcus rosae

XML Treatment for Sirococcus rossmaniae

XML Treatment for Sirococcus sanguisorbae

XML Treatment for Sirococcus saprophyticus

XML Treatment for Sirococcus silvicola

XML Treatment for Sirococcus smithogilvyi

XML Treatment for Sirococcus taishanensis

XML Treatment for Sirococcus tormentillae

XML Treatment for Sirococcus xishuangbannaensis

XML Treatment for Sirococcus xunwuensis

XML Treatment for Sirococcus yunnanensis

## Additional information

### Use of AI

No use of AI was reported.

### Adherence to national and international regulations

All the fungal strains used in this study have been legally obtained, respecting the Convention on Biological Diversity (Rio Convention).

### Author ORCIDs

Ning Jiang https://orcid.org/0000-0002-9656-8500

Nalin N. Wijayawardene https://orcid.org/0000-0003-0522-5498

### Data availability

All of the data that support the findings of this study are available in the main text or Supplementary Information.

## Supplementary materials

Supplementary material 1 Isolates and accession numbers of sequences used in the phylogenetic analyses of *Sirococcus* Data type docx https://binary.pensoft.net/file/1560187This dataset is made available under the Open Database License (http://opendatacommons.org/licenses/odbl/1.0/). The Open Database License (ODbL) is a license agreement intended to allow users to freely share, modify, and use this Dataset while maintaining this same freedom for others, provided that the original source and author(s) are credited. Ning Jiang, Nalin N. Wijayawardene, Han Xue, Yong Li

Supplementary material 2 An alignment used in this study Data type fas https://binary.pensoft.net/file/1560188This dataset is made available under the Open Database License (http://opendatacommons.org/licenses/odbl/1.0/). The Open Database License (ODbL) is a license agreement intended to allow users to freely share, modify, and use this Dataset while maintaining this same freedom for others, provided that the original source and author(s) are credited. Ning Jiang, Nalin N. Wijayawardene, Han Xue, Yong Li

Supplementary material 3 Single-gene phylograms of *Sirococcus* Data type zip https://binary.pensoft.net/file/1560189This dataset is made available under the Open Database License (http://opendatacommons.org/licenses/odbl/1.0/). The Open Database License (ODbL) is a license agreement intended to allow users to freely share, modify, and use this Dataset while maintaining this same freedom for others, provided that the original source and author(s) are credited. Ning Jiang, Nalin N. Wijayawardene, Han Xue, Yong Li
