# Supplementary material for: Reevaluating Sirococcus: synonymizing Gnomoniopsis and elucidating the life cycle of S. daii
Source: IMA Fungus. 2026 Mar 12;17:e186049. doi: 10.3897/imafungus.17.186049 (PMC13003334; doi:10.3897/imafungus.17.186049)
Supplement: Supplementary material 1 — Isolates and accession numbers of sequences used in the phylogenetic analyses of Sirococcus [file imafungus-17-e186049-s001.docx]

**Table S1** Isolates and accession numbers of sequences used in the phylogenetic analyses of *Sirococcus*.

| **Current name** | **Previous name** | **Country** | **Host** | **Host Family** | **Strain** | **GenBank Accession Number** | | | **References** |
| --- | --- | --- | --- | --- | --- | --- | --- | --- | --- |
|  |  |  |  |  |  | **ITS** | ***tef1*** | ***tub2*** |  |
| *Apiognomonia errabunda* |  | Switzerland | *Fagus sylvatica* | Fagaceae | AR 2813 | DQ313525 | DQ313565 | DQ862014 | Sogonov et al. 2007 |
| *Discula destructiva* |  | USA | *Cornus nuttallii* | Cornaceae | CBS 109771 | EF512464 | EF512526 | NA | Rossman et al. 2008 |
| *Discula destructiva* |  | USA | *Cornus florida* | Cornaceae | AR 2817 | EF512465 | EF512527 | NA | Rossman et al. 2008 |
| *Gnomonia gnomon* |  | Italy | *Corylus avellana* | Betulaceae | CBS 199.53 | DQ491518 | EU221885 | EU219148 | James et al. 2006 |
| *Sirococcus alderdunensis* | *Gnomoniopsis alderdunensis* | USA | *Rubus pedatus* | Rosaeace | CBS 125679 | GU320826 | GU320813 | GU320788 | Walker et al. 2010 |
| *Sirococcus alderdunensis* | *Gnomoniopsis alderdunensis* | USA | *Rubus parviflorus* | Rosaeace | CBS 125680* | GU320825 | GU320801 | GU320787 | Walker et al. 2010 |
| *Sirococcus alderdunensis* | *Gnomoniopsis alderdunensis* | USA | *Rubus parviflorus* | Rosaeace | CBS 125681 | GU320827 | GU320802 | GU320789 | Walker et al. 2010 |
| *Sirococcus angolensis* | *Gnomoniopsis angolensis* | Angola | Unknown | Unknown | CBS 145057* | MK047428 | NA | NA | Crous et al. 2018 |
| *Sirococcus annonae* | *Gnomoniopsis annonae* | China | *Annona montana* | Annonaceae | CGMCC 3.28195* | PQ325623 | PQ336654 | PQ336678 | Zhang et al. 2025 |
| *Sirococcus annonae* | *Gnomoniopsis annonae* | China | *Annona montana* | Annonaceae | SAUCC 6333 | PQ325624 | PQ336655 | PQ336679 | Zhang et al. 2025 |
| *Sirococcus annonae* | *Gnomoniopsis annonae* | China | *Annona montana* | Annonaceae | CGMCC 3.28194 | PQ325625 | PQ336656 | PQ336680 | Zhang et al. 2025 |
| *Sirococcus annonae* | *Gnomoniopsis annonae* | China | *Annona montana* | Annonaceae | SAUCC 6423 | PQ325626 | PQ336657 | PQ336681 | Zhang et al. 2025 |
| *Sirococcus castaneae* |  | Switzerland | *Castanea sativa* | Fagaceae | CBS 142041 | KX929744 | KX929710 | KX958443 | Meyer et al. 2017 |
| *Sirococcus castanopsidis* | *Gnomoniopsis castanopsidis* | China | *Castanopsis hystrix* | Fagaceae | CFCC 54437* | MZ902909 | MZ936385 | NA | Jiang et al. 2021b |
| *Sirococcus castanopsidis* | *Gnomoniopsis castanopsidis* | China | *Castanopsis hystrix* | Fagaceae | CFCC 55878 | MZ902910 | MZ936386 | NA | Jiang et al. 2021b |
| *Sirococcus chamaemori* | *Gnomoniopsis chamaemori* | Finland | *Rubus chamaemorus* | Rosaeace | CBS 804.79 | GU320817 | GU320809 | GU320777 | Walker et al. 2010 |
| *Sirococcus chinensis* | *Gnomoniopsis chinensis* | China | *Castanea mollissima* | Fagaceae | CFCC 52286* | MG866032 | MH545370 | MH545366 | Jiang et al. 2020 |
| *Sirococcus chinensis* | *Gnomoniopsis chinensis* | China | *Castanea mollissima* | Fagaceae | CFCC 52287 | MG866033 | MH545371 | MH545367 | Jiang et al. 2020 |
| *Sirococcus chinensis* | *Gnomoniopsis chinensis* | China | *Castanea mollissima* | Fagaceae | CFCC 52288 | MG866034 | MH545372 | MH545368 | Jiang et al. 2020 |
| *Sirococcus chinensis* | *Gnomoniopsis chinensis* | China | *Castanea mollissima* | Fagaceae | CFCC 52289 | MG866035 | MH545373 | MH545369 | Jiang et al. 2020 |
| *Sirococcus clavulatus* | *Gnomoniopsis clavulata* | USA | *Quercus falcata* | Fagaceae | CBS 121255 | EU254818 | GU320807 | EU219211 | Sogonov et al. 2008 |
| *Sirococcus comari* | *Gnomoniopsis comari* | Finland | *Comarum palustre* | Rosaeace | CBS 806.79 | EU254821 | GU320810 | EU219156 | Sogonov et al. 2008 |
| *Sirococcus comari* | *Gnomoniopsis comari* | Finland | *Comarum palustre* | Rosaeace | CBS 807.79 | EU254822 | GU320814 | GU320779 | Sogonov et al. 2008 |
| *Sirococcus comari* | *Gnomoniopsis comari* | Switzerland | *Comarum palustre* | Rosaeace | CBS 809.79 | EU254823 | GU320794 | GU320778 | Sogonov et al. 2008 |
| *Sirococcus conigenus* |  | Austria | *Picea abies* | Pinaceae | CBS 101225 | EF512481 | EF512543 | NA | Rossman et al. 2008 |
| *Sirococcus conigenus* |  | Canada | *Pinus contorta* | Pinaceae | CBS 119616 | EF512468 | EF512530 | NA | Rossman et al. 2008 |
| *Sirococcus conigenus* |  | Finland | *Picea abies* | Pinaceae | CBS 119615* | EF512470 | EF512532 | NA | Rossman et al. 2008 |
| *Sirococcus conigenus* |  | Canada | *Larix laricina* | Pinaceae | DAOM 191767 | EF512483 | EF512545 | NA | Rossman et al. 2008 |
| *Sirococcus conigenus* |  | Canada | *Larix laricina* | Pinaceae | DAOM 191768 | EF512484 | EF512546 | NA | Rossman et al. 2008 |
| *Sirococcus conigenus* |  | Canada | *Larix laricina* | Pinaceae | CBS 119602 | EF512467 | EF512529 | NA | Rossman et al. 2008 |
| *Sirococcus conigenus* |  | Germany | *Picea pungens* | Pinaceae | CBS 113.75 | EF512482 | EF512544 | EU219129 | Rossman et al. 2008 |
| *Sirococcus conigenus* |  | USA | *Cedrus deodara* | Pinaceae | AR3965 | EF512477 | EF512539 | NA | Rossman et al. 2008 |
| *Sirococcus daii* | *Gnomoniopsis daii* | China | *Castanea mollissima* | Fagaceae | CFCC 54043* | MN598671 | MN605519 | MN605517 | Jiang and Tian 2019 |
| *Sirococcus daii* | *Gnomoniopsis daii* | China | *Castanea mollissima* | Fagaceae | CMF002B | MN598672 | MN605520 | MN605518 | Jiang and Tian 2019 |
| *Sirococcus daii* | *Gnomoniopsis daii* | China | *Quercus aliena* | Fagaceae | CFCC 55517 | MZ902911 | MZ936387 | MZ936403 | Jiang et al. 2021b |
| *Sirococcus daii* | *Gnomoniopsis daii* | China | *Quercus aliena* | Fagaceae | CFCC 55294B | MZ902912 | MZ936388 | MZ936404 | Jiang et al. 2021b |
| *Sirococcus daii* | *Gnomoniopsis daii* | China | *Castanopsis fargesii* | Fagaceae | CGMCC 3.28193 | PQ325627 | PQ336658 | PQ336682 | Zhang et al. 2025 |
| *Sirococcus daii* | *Gnomoniopsis daii* | China | *Castanopsis fargesii* | Fagaceae | SAUCC 0153 | PQ325628 | PQ336659 | PQ336683 | Zhang et al. 2025 |
| *Sirococcus daii* | *Gnomoniopsis daii* | China | *Castanea mollissima* | Fagaceae | CFCC 54345 | MW208113 | MW227345 | MW218543 | Jiang et al. 2021a |
| *Sirococcus daii* | *Gnomoniopsis daii* | China | *Castanea mollissima* | Fagaceae | ZY10-1 | MW208114 | MW227346 | MW218544 | Jiang et al. 2021a |
| *Sirococcus daii* | *Gnomoniopsis daii* | China | *Castanea mollissima* | Fagaceae | ZY10-3 | MW208115 | MW227347 | MW218545 | Jiang et al. 2021a |
| *Sirococcus daii* | *Gnomoniopsis daii* | China | *Castanea mollissima* | Fagaceae | ZY12A | MW208116 | MW227348 | MW218546 | Jiang et al. 2021a |
| *Sirococcus daii* | *Gnomoniopsis daii* | China | *Castanea mollissima* | Fagaceae | ZX14-1 | MW208117 | MW227349 | MW218547 | Jiang et al. 2021a |
| *Sirococcus daii* |  | China | *Castanea mollissima* | Fagaceae | CFCC 72005 | PX982824 | PX981890 | PX981902 | This study |
| *Sirococcus daii* |  | China | *Castanea mollissima* | Fagaceae | CFCC 72006 | PX982825 | PX981891 | PX981903 | This study |
| *Sirococcus daii* |  | China | *Castanea mollissima* | Fagaceae | CFCC 71611 | PX982826 | PX981892 | PX981904 | This study |
| *Sirococcus daii* |  | China | *Castanea mollissima* | Fagaceae | CFCC 71612 | PX982827 | PX981893 | PX981905 | This study |
| *Sirococcus daii* |  | China | *Castanea henryi* | Fagaceae | CFCC 71935 | PX982828 | PX981894 | PX981906 | This study |
| *Sirococcus daii* |  | China | *Castanea henryi* | Fagaceae | CFCC 71938 | PX982829 | PX981895 | PX981907 | This study |
| *Sirococcus daii* |  | China | *Castanea mollissima* | Fagaceae | CFCC 71970 | PX982830 | PX981896 | PX981908 | This study |
| *Sirococcus diaoluoshanensis* | *Gnomoniopsis diaoluoshanensis* | China | *Castanopsis chinensis* | Fagaceae | SAUCC DL0963* | ON753744 | ON759769 | ON759777 | Wang et al. 2022 |
| *Sirococcus diaoluoshanensis* | *Gnomoniopsis diaoluoshanensis* | China | *Castanopsis chinensis* | Fagaceae | SAUCC DL0964 | ON753743 | ON759768 | ON759776 | Wang et al. 2022 |
| *Sirococcus euryae* | *Gnomoniopsis euryae* | China | *Eurya nitida* | Theaceae | CGMCC 3.28234* | PQ325629 | PQ336660 | PQ336684 | Zhang et al. 2025 |
| *Sirococcus euryae* | *Gnomoniopsis euryae* | China | *Eurya nitida* | Theaceae | SAUCC 3977 | PQ325630 | PQ336661 | PQ336685 | Zhang et al. 2025 |
| *Sirococcus fagacearum* | *Gnomoniopsis fagacearum* | China | *Lithocarpus glaber* | Fagaceae | CFCC 54316* | MZ902916 | MZ936392 | MZ936408 | Jiang et al. 2021b |
| *Sirococcus flavus* | *Gnomoniopsis flava* | China | *Castanopsis carlesii* | Fagaceae | CFCC 71563* | PV257808 | PV268106 | PV339811 | Li et al. 2025 |
| *Sirococcus flavus* | *Gnomoniopsis flava* | China | *Castanopsis carlesii* | Fagaceae | CFCC 71566 | PV257809 | PV268107 | PV339812 | Li et al. 2025 |
| *Sirococcus flavus* | *Gnomoniopsis flava* | China | *Castanopsis carlesii* | Fagaceae | CFCC 71567 | PV257810 | PV268108 | PV339813 | Li et al. 2025 |
| *Sirococcus fragariae* | *Gnomoniopsis fragariae* | USA | *Fragaria vesca* | Rosaeace | CBS 121226 | EU254824 | GU320792 | EU219144 | Sogonov et al. 2008 |
| *Sirococcus fragariae* | *Gnomoniopsis fragariae* | France | *Fragaria* sp*.* | Rosaeace | CBS 208.34 | EU254826 | GU320808 | EU219149 | Sogonov et al. 2008 |
| *Sirococcus fragariae* | *Gnomoniopsis fragariae* | USA | *Fragaria* sp*.* | Rosaeace | CBS 125671 | GU320816 | GU320793 | GU320776 | Sogonov et al. 2008 |
| *Sirococcus fujianensis* | *Gnomoniopsis fujianensis* | China | *Castanopsis chinensis* | Fagaceae | CGMCC 3.28229* | PQ325641 | PQ336672 | PQ336696 | Zhang et al. 2025 |
| *Sirococcus fujianensis* | *Gnomoniopsis fujianensis* | China | *Castanopsis chinensis* | Fagaceae | SAUCC 1260 | PQ325642 | PQ336673 | PQ336697 | Zhang et al. 2025 |
| *Sirococcus guangdongensis* | *Gnomoniopsis guangdongensis* | China | *Castanopsis fargesii* | Fagaceae | CFCC 54443* | MZ902918 | MZ936394 | MZ936410 | Jiang et al. 2021b |
| *Sirococcus guangdongensis* | *Gnomoniopsis guangdongensis* | China | *Castanopsis fargesii* | Fagaceae | CFCC 54331 | MZ902919 | MZ936395 | MZ936411 | Jiang et al. 2021b |
| *Sirococcus guangdongensis* | *Gnomoniopsis guangdongensis* | China | *Castanopsis fargesii* | Fagaceae | CFCC 54282 | MZ902920 | MZ936396 | MZ936412 | Jiang et al. 2021b |
| *Sirococcus guttulatus* | *Gnomoniopsis guttulata* | Bulgaria | *Agrimonia eupatoria* | Rosaeace | MS 0312 | EU254812 | NA | NA | Sogonov et al. 2008 |
| *Sirococcus hainanensis* | *Gnomoniopsis hainanensis* | China | *Castanopsis hainanensis* | Fagaceae | CFCC 54376* | MZ902921 | MZ936397 | MZ936413 | Jiang et al. 2021b |
| *Sirococcus hainanensis* | *Gnomoniopsis hainanensis* | China | *Castanopsis hainanensis* | Fagaceae | CFCC 55877 | MZ902922 | MZ936398 | MZ936414 | Jiang et al. 2021b |
| *Sirococcus idaeicola* | *Gnomoniopsis idaeicola* | USA | *Rubus* sp*.* | Rosaeace | CBS 125672 | GU320823 | GU320797 | GU320781 | Walker et al. 2010 |
| *Sirococcus idaeicola* | *Gnomoniopsis idaeicola* | USA | *Rubus pedatus* | Rosaeace | CBS 125673 | GU320824 | GU320798 | GU320782 | Walker et al. 2010 |
| *Sirococcus idaeicola* | *Gnomoniopsis idaeicola* | France | *Rubus* sp. | Rosaeace | CBS 125674* | GU320820 | GU320796 | GU320780 | Walker et al. 2010 |
| *Sirococcus idaeicola* | *Gnomoniopsis idaeicola* | USA | *Rubus procerus* | Rosaeace | CBS 125675 | GU320822 | GU320799 | GU320783 | Walker et al. 2010 |
| *Sirococcus idaeicola* | *Gnomoniopsis idaeicola* | USA | *Rubus procerus* | Rosaeace | CBS 125676 | GU320821 | GU320811 | GU320784 | Walker et al. 2010 |
| *Sirococcus juglandis* | *Gnomoniopsis juglandis* | China | *Juglans regia* | Juglandaceae | CGMCC 3.28233* | PQ325637 | PQ336668 | PQ336692 | Zhang et al. 2025 |
| *Sirococcus juglandis* | *Gnomoniopsis juglandis* | China | *Juglans regia* | Juglandaceae | SAUCC 2375 | PQ325638 | PQ336669 | PQ336693 | Zhang et al. 2025 |
| *Sirococcus lanceolatae* | *Gnomoniopsis lanceolata* | China | *Phoebe lanceolata* | Lauraceae | CGMCC 3.28232* | PQ325639 | PQ336670 | PQ336694 | Zhang et al. 2025 |
| *Sirococcus lanceolatae* | *Gnomoniopsis lanceolata* | China | *Phoebe lanceolata* | Lauraceae | SAUCC 1277 | PQ325640 | PQ336671 | PQ336695 | Zhang et al. 2025 |
| *Sirococcus lithocarpi* | *Gnomoniopsis lithocarpi* | China | *Lithocarpus fohaiensis* | Fagaceae | SAUCC YN0743* | ON753749 | ON759765 | ON759783 | Wang et al. 2022 |
| *Sirococcus lithocarpi* | *Gnomoniopsis lithocarpi* | China | *Lithocarpus fohaiensis* | Fagaceae | SAUCC YN0742 | ON753750 | ON759764 | ON759782 | Wang et al. 2022 |
| *Sirococcus macounii* | *Gnomoniopsis macounii* | USA | *Spiraea* sp. | Rosaeace | CBS 121468 | EU254762 | GU320804 | EU219126 | Sogonov et al. 2008 |
| *Sirococcus melastomatis* | *Gnomoniopsis melastomatis* | China | *Melastoma candidum* | Melastomataceae | CGMCC 3.28231* | PQ325631 | PQ336662 | PQ336686 | Zhang et al. 2025 |
| *Sirococcus melastomatis* | *Gnomoniopsis melastomatis* | China | *Melastoma candidum* | Melastomataceae | SAUCC 0031 | PQ325632 | PQ336663 | PQ336687 | Zhang et al. 2025 |
| *Sirococcus mengyinensis* | *Gnomoniopsis mengyinensis* | China | *Castanea mollissima* | Fagaceae | SAUCC MY0293* | ON753741 | ON759766 | ON759774 | Wang et al. 2022 |
| *Sirococcus mengyinensis* | *Gnomoniopsis mengyinensis* | China | *Castanea mollissima* | Fagaceae | SAUCC MY0296 | ON753742 | ON759767 | ON759775 | Wang et al. 2022 |
| *Sirococcus mengyinensis* |  | China | *Castanea mollissima* | Fagaceae | CFCC 71876 | PX982831 | PX981897 | PX981909 | This study |
| *Sirococcus mengyinensis* |  | China | *Castanea mollissima* | Fagaceae | CFCC 71877 | PX982832 | PX981898 | PX981910 | This study |
| *Sirococcus mengyinensis* |  | China | *Castanea mollissima* | Fagaceae | CFCC 71908 | PX982833 | PX981899 | PX981911 | This study |
| *Sirococcus occultus* | *Gnomoniopsis occulta* | USA | *Potentilla* sp. | Rosaeace | CBS 125677 | GU320828 | GU320812 | GU320785 | Walker et al. 2010 |
| *Sirococcus occultus* | *Gnomoniopsis occulta* | USA | *Potentilla* sp. | Rosaeace | CBS 125678* | GU320829 | GU320800 | GU320786 | Walker et al. 2010 |
| *Sirococcus paraclavulatus* | *Gnomoniopsis paraclavulata* | USA | *Quercus alba* | Fagaceae | CBS 123202 | GU320830 | GU320815 | GU320775 | Walker et al. 2010 |
| *Sirococcus piceicola* |  | Canada | *Picea sitchensis* | Pinaceae | CBS 119620* | EF512480 | EF512542 | EU219130 | Rossman et al. 2008 |
| *Sirococcus piceicola* |  | Canada | *Picea glauca* | Pinaceae | CBS 119625 | EF512475 | EF512537 | NA | Rossman et al. 2008 |
| *Sirococcus piceicola* |  | Switzerland | *Picea abies* | Pinaceae | CBS 119621 | EF512474 | EF512536 | NA | Rossman et al. 2008 |
| *Sirococcus quercus* |  | USA | *Quercus sp.* | Fagaceae | CBS 142126 | KY173465 | NA | NA | Crous et al. 2013 |
| *Sirococcus racemulus* | *Gnomoniopsis racemula* | USA | *Chamerion angustifolium* | Onagraceae | CBS 121469* | EU254841 | GU320803 | EU219125 | Sogonov et al. 2008 |
| *Sirococcus rosae* | *Gnomoniopsis rosae* | New Zealand | *Rosa* sp*.* | Rosaeace | CBS 145 085* | MK047451 | NA | NA | Crous et al. 2018 |
| *Sirococcus rosae* |  | China | *Rosa chinensis* | Rosaeace | CFCC 72007 | PX982834 | PX981900 | PX981912 | This study |
| *Sirococcus rossmaniae* | *Gnomoniopsis rossmaniae* | China | *Castanopsis hainanensis* | Fagaceae | CFCC 54307* | MZ902923 | MZ936399 | MZ936415 | Jiang et al. 2021b |
| *Sirococcus rossmaniae* | *Gnomoniopsis rossmaniae* | China | *Castanopsis hainanensis* | Fagaceae | CFCC 55876 | MZ902924 | MZ936400 | MZ936416 | Jiang et al. 2021b |
| *Sirococcus sanguisorbae* | *Gnomoniopsis sanguisorbae* | Switzerland | *Sanguisorba minor* | Rosaeace | CBS 858.79 | GU320818 | GU320805 | GU320790 | Walker et al. 2010 |
| *Sirococcus saprophyticus* | *Gnomoniopsis saprophytica* | China | *Melastoma candidum* | Melastomataceae | SAUCC 3040 | PQ325633 | PQ336664 | PQ336688 | Zhang et al. 2025 |
| *Sirococcus saprophyticus* | *Gnomoniopsis saprophytica* | China | *Melastoma candidum* | Melastomataceae | SAUCC 0051 | PQ325634 | PQ336665 | PQ336689 | Zhang et al. 2025 |
| *Sirococcus saprophyticus* | *Gnomoniopsis saprophytica* | China | Unknown | Unknown | CGMCC 3.28192* | PQ325633 | PQ336664 | PQ336688 | Zhang et al. 2025 |
| *Sirococcus saprophyticus* | *Gnomoniopsis saprophytica* | China | Unknown | Unknown | SAUCC 3342 | PQ325634 | PQ336665 | PQ336689 | Zhang et al. 2025 |
| *Sirococcus silvicola* | *Gnomoniopsis silvicola* | China | *Castanopsis hystrix* | Fagaceae | CFCC 54304 | MZ902925 | MZ936401 | MZ936417 | Jiang et al. 2021b |
| *Sirococcus silvicola* | *Gnomoniopsis silvicola* | China | *Quercus serrata* | Fagaceae | CFCC 54418* | MZ902926 | MZ936402 | MZ936418 | Jiang et al. 2021b |
| *Sirococcus silvicola* |  | China | *Castanopsis carlesii* | Fagaceae | CFCC 71885 | PX982835 | PX981901 | PX981913 | This study |
| *Sirococcus smithogilvyi* | *Gnomoniopsis smithogilvyi* | Australia | *Castanea* sp. | Fagaceae | CBS 130190* | JQ910642 | KR072534 | JQ910639 | Crous et al. 2012 |
| *Sirococcus smithogilvyi* | *Gnomoniopsis smithogilvyi* | Australia | *Castanea* sp. | Fagaceae | CBS 130189 | JQ910644 | KR072535 | JQ910641 | Crous et al. 2012 |
| *Sirococcus smithogilvyi* | *Gnomoniopsis smithogilvyi* | Australia | *Castanea* sp. | Fagaceae | CBS 130188 | JQ910643 | KR072536 | JQ910640 | Crous et al. 2012 |
| *Sirococcus smithogilvyi* | *Gnomoniopsis smithogilvyi* | Italy | *Castanea sativa* | Fagaceae | MUT 401 | HM142946 | KR072537 | KR072532 | Visentin et al. 2012 |
| *Sirococcus smithogilvyi* | *Gnomoniopsis smithogilvyi* | New Zealand | *Castanea sativa* | Fagaceae | MUT 411 | HM142948 | KR072538 | KR072533 | Visentin et al. 2012 |
| *Sirococcus taishanensis* | *Gnomoniopsis taishanensis* | China | *Castanea mollissima* | Fagaceae | CGMCC 3.28230* | PQ325645 | PQ336676 | PQ336700 | Zhang et al. 2025 |
| *Sirococcus taishanensis* | *Gnomoniopsis taishanensis* | China | *Castanea mollissima* | Fagaceae | SAUCC 1033 | PQ325646 | PQ336677 | PQ336701 | Zhang et al. 2025 |
| *Sirococcus tormentillae* | *Gnomoniopsis tormentillae* | Switzerland | *Potentilla* sp. | Rosaeace | CBS 904.79 | EU254856 | GU320795 | EU219165 | Sogonov et al. 2008 |
| *Sirococcus tsugae* |  | USA | *Tsuga mertensiana* | Pinaceae | CBS 119626 | EU199203 | EF512534 | EU219140 | Rossman et al. 2008 |
| *Sirococcus tsugae* |  | USA | *Tsuga mertensiana* | Pinaceae | CBS 119619 | EF512473 | EF512535 | NA | Rossman et al. 2008 |
| *Sirococcus tsugae* |  | USA | *Tsuga heterophylla* | Pinaceae | CBS 119624 | EF512471 | EF512533 | NA | Rossman et al. 2008 |
| *Sirococcus tsugae* |  | USA | *Tsuga heterophylla* | Pinaceae | CBS 119617 | EF512466 | EF512528 | NA | Rossman et al. 2008 |
| *Sirococcus tsugae* |  | USA | *Cedrus deodara* | Pinaceae | CBS 119627 | EF512478 | EF512540 | EU219143 | Rossman et al. 2008 |
| *Sirococcus tsugae* |  | USA | *Cedrus atlantica* | Pinaceae | CBS 119623 | EF512476 | EF512538 | NA | Rossman et al. 2008 |
| *Sirococcus tsugae* |  | USA | *Cedrus deodara* | Pinaceae | CBS 119618 | EF512479 | EF512541 | NA | Rossman et al. 2008 |
| *Sirococcus tsugae* |  | Canada | *Tsuga heterophylla* | Pinaceae | CBS 119622 | EF512469 | EF512531 | NA | Rossman et al. 2008 |
| *Sirococcus tsugae* |  | NA | NA | NA | CBS 128356 | JF514853 | JF514834 | JF514844 | Walker et al. 2012b |
| *Sirococcus xishuangbannaensis* | *Gnomoniopsis xishuangbannaensis* | China | *Castanea mollissima* | Fagaceae | CGMCC 3.25980* | PQ325643 | PQ336674 | PQ336698 | Zhang et al. 2025 |
| *Sirococcus xishuangbannaensis* | *Gnomoniopsis xishuangbannaensis* | China | *Castanea mollissima* | Fagaceae | CGMCC 3.25979 | PQ325644 | PQ336675 | PQ336699 | Zhang et al. 2025 |
| *Sirococcus xunwuensis* | *Gnomoniopsis xunwuensis* | China | *Castanopsis fissa* | Fagaceae | CFCC 53115* | MK432667 | MK578141 | MK578067 | Yang et al. 2020 |
| *Sirococcus xunwuensis* | *Gnomoniopsis xunwuensis* | China | *Castanopsis fissa* | Fagaceae | CFCC 53116 | MK432668 | MK578142 | MK578068 | Yang et al. 2020 |
| *Sirococcus yunnanensis* | *Gnomoniopsis yunnanensis* | China | *Castanea mollissima* | Fagaceae | SAUCC YN1659* | ON753746 | ON759771 | ON759779 | Wang et al. 2022 |
| *Sirococcus yunnanensis* | *Gnomoniopsis yunnanensis* | China | *Castanea mollissima* | Fagaceae | SAUCC YN1657 | ON753747 | ON759772 | ON759780 | Wang et al. 2022 |

Note. Ex-type strains are marked with *, and NA means not available.
