# Supplementary figures and images for: Reevaluating Sirococcus: synonymizing Gnomoniopsis and elucidating the life cycle of S. daii
Source: IMA Fungus. 2026 Mar 12;17:e186049. doi: 10.3897/imafungus.17.186049 (PMC13003334; doi:10.3897/imafungus.17.186049)

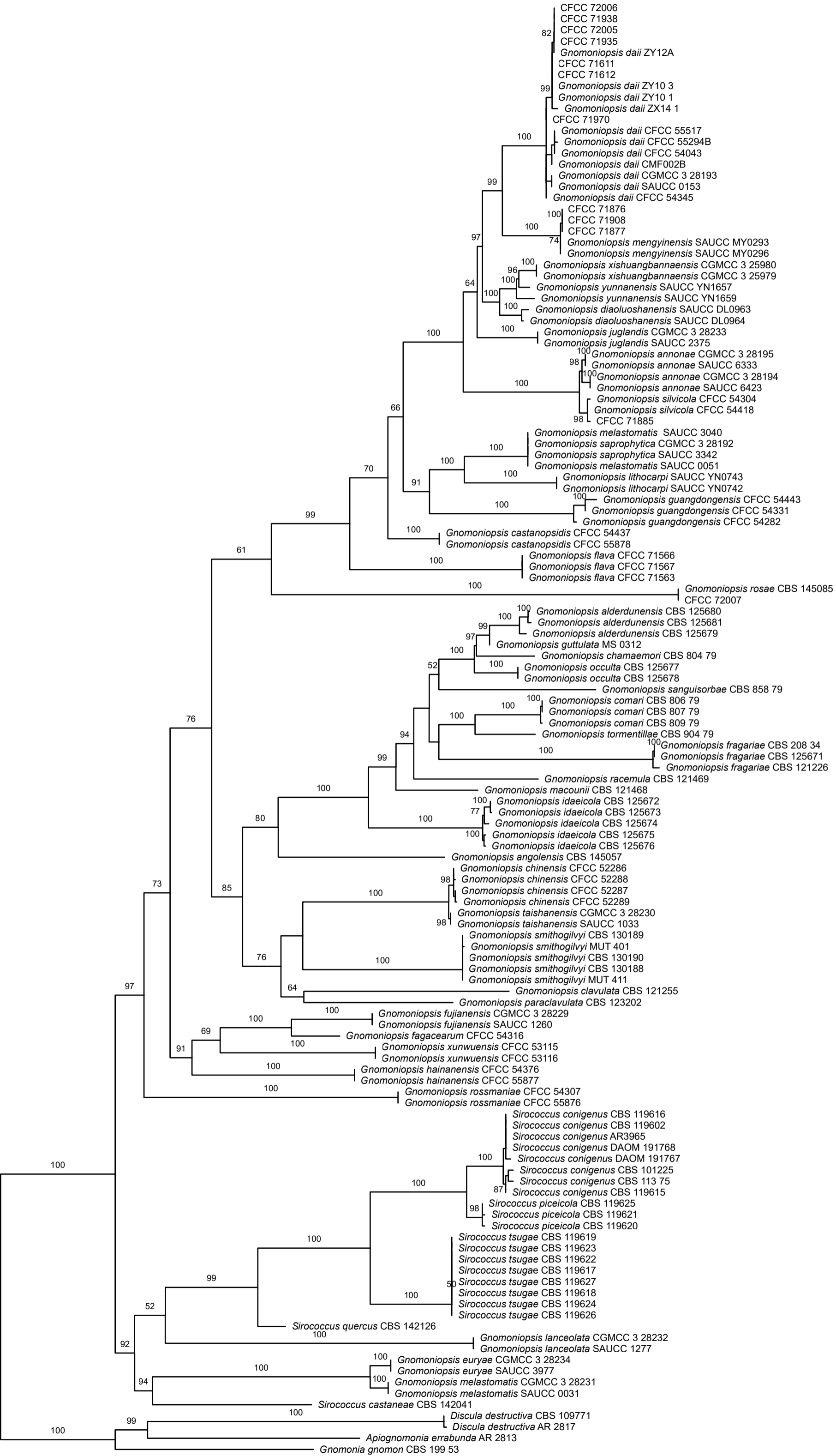

0.04

Supplement: Supplementary material 3 — Single-gene phylograms of Sirococcus [file imafungus-17-e186049-s003.zip › 186049_0R-1-A_ITS-tef_phylogeny.pdf]

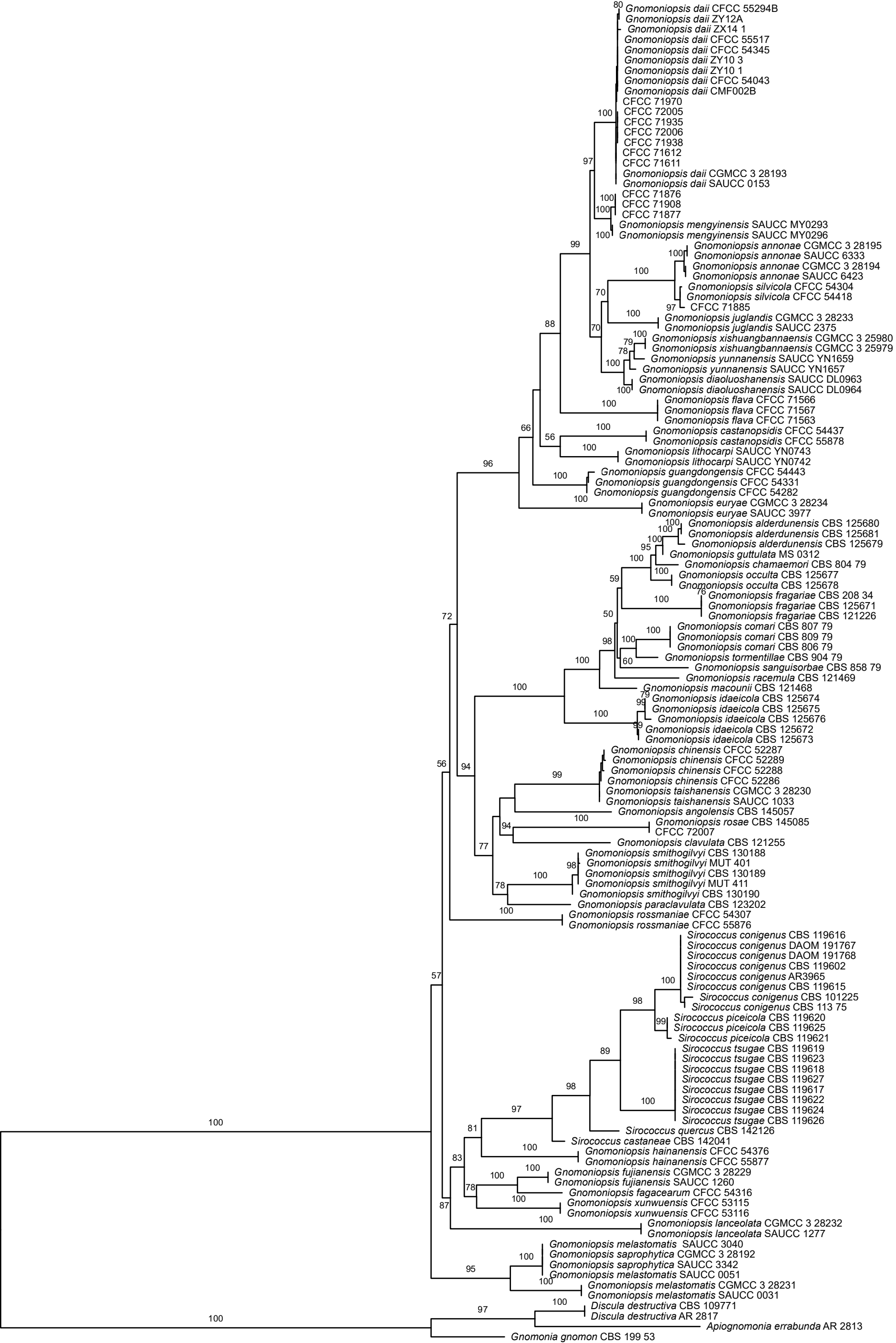

0.09

Supplement: Supplementary material 3 — Single-gene phylograms of Sirococcus [file imafungus-17-e186049-s003.zip › 186049_0R-1-A_ITS-tub_phylogeny.pdf]

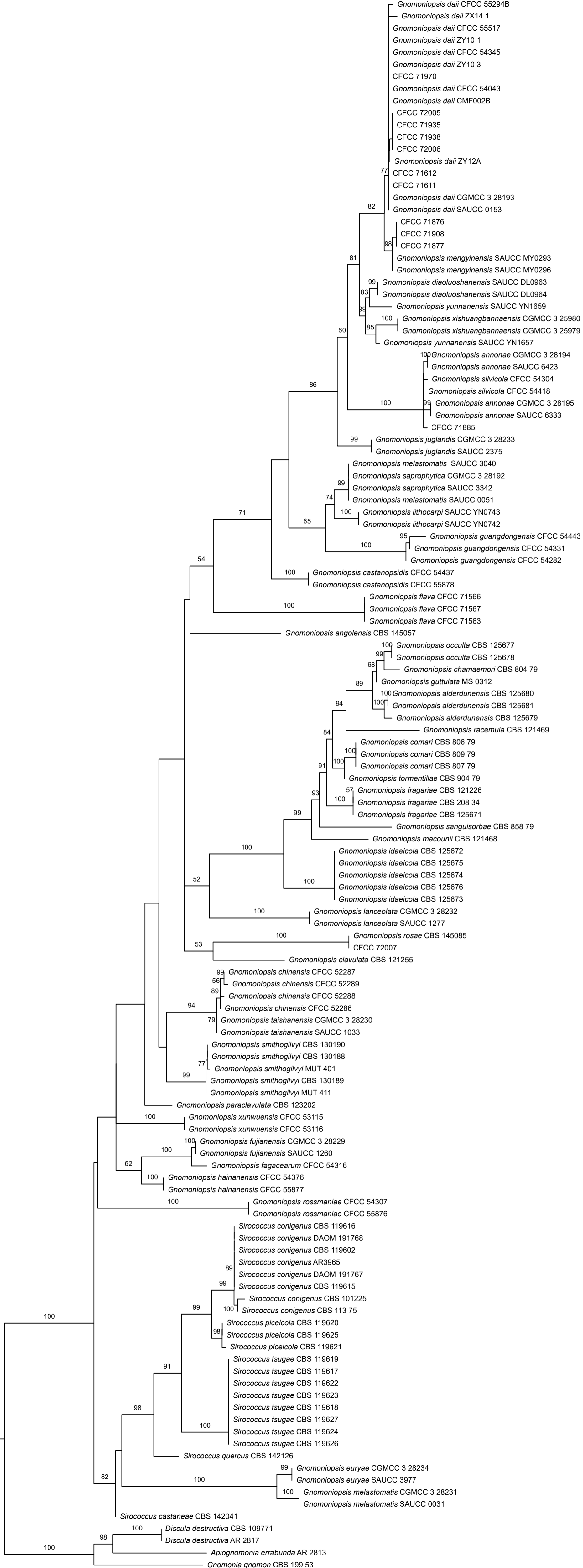

0.04

Supplement: Supplementary material 3 — Single-gene phylograms of Sirococcus [file imafungus-17-e186049-s003.zip › 186049_0R-1-A_ITS_phylogeny.pdf]

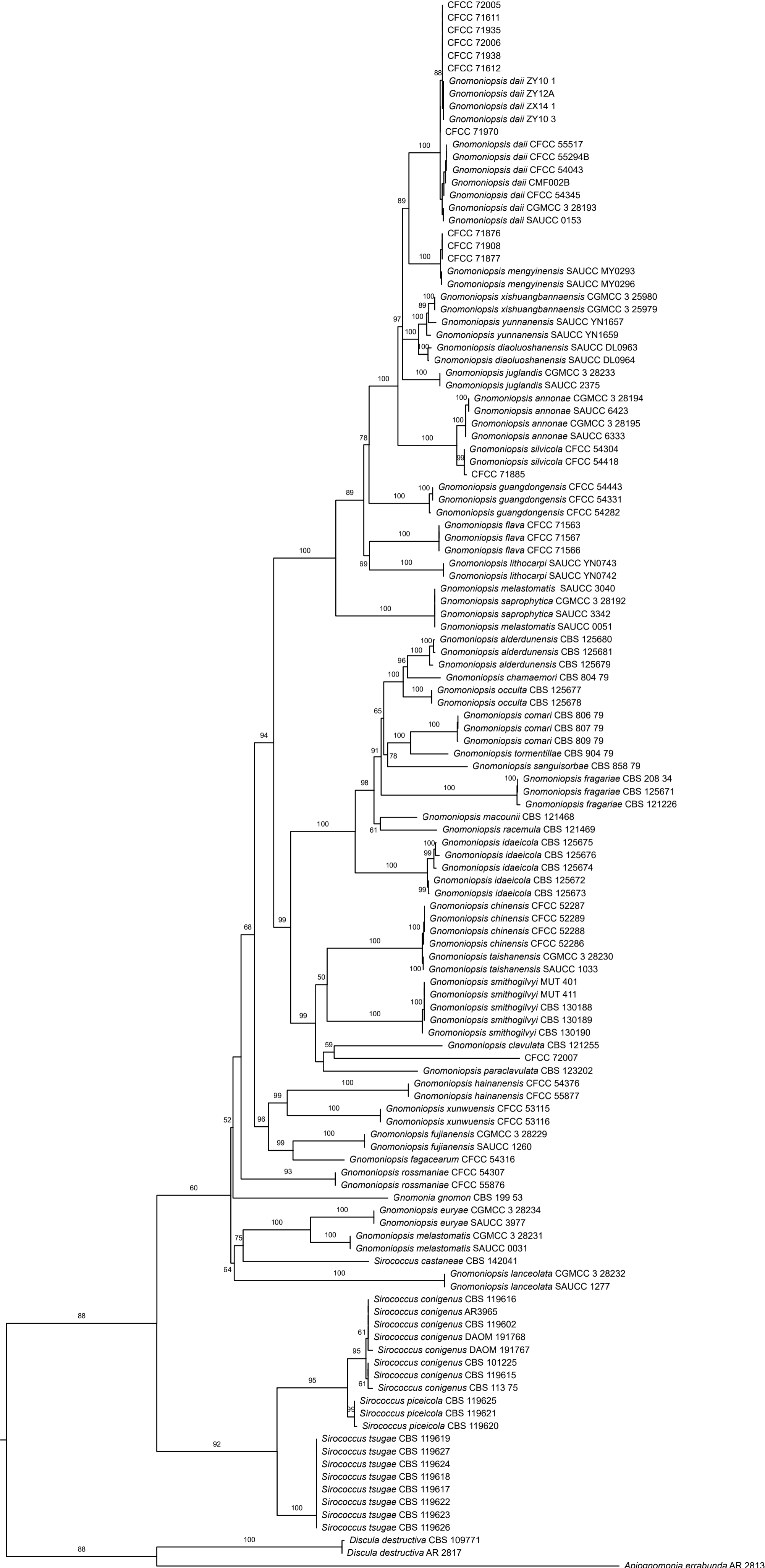

0.07

Supplement: Supplementary material 3 — Single-gene phylograms of Sirococcus [file imafungus-17-e186049-s003.zip › 186049_0R-1-A_tef-tub_phylogeny.pdf]

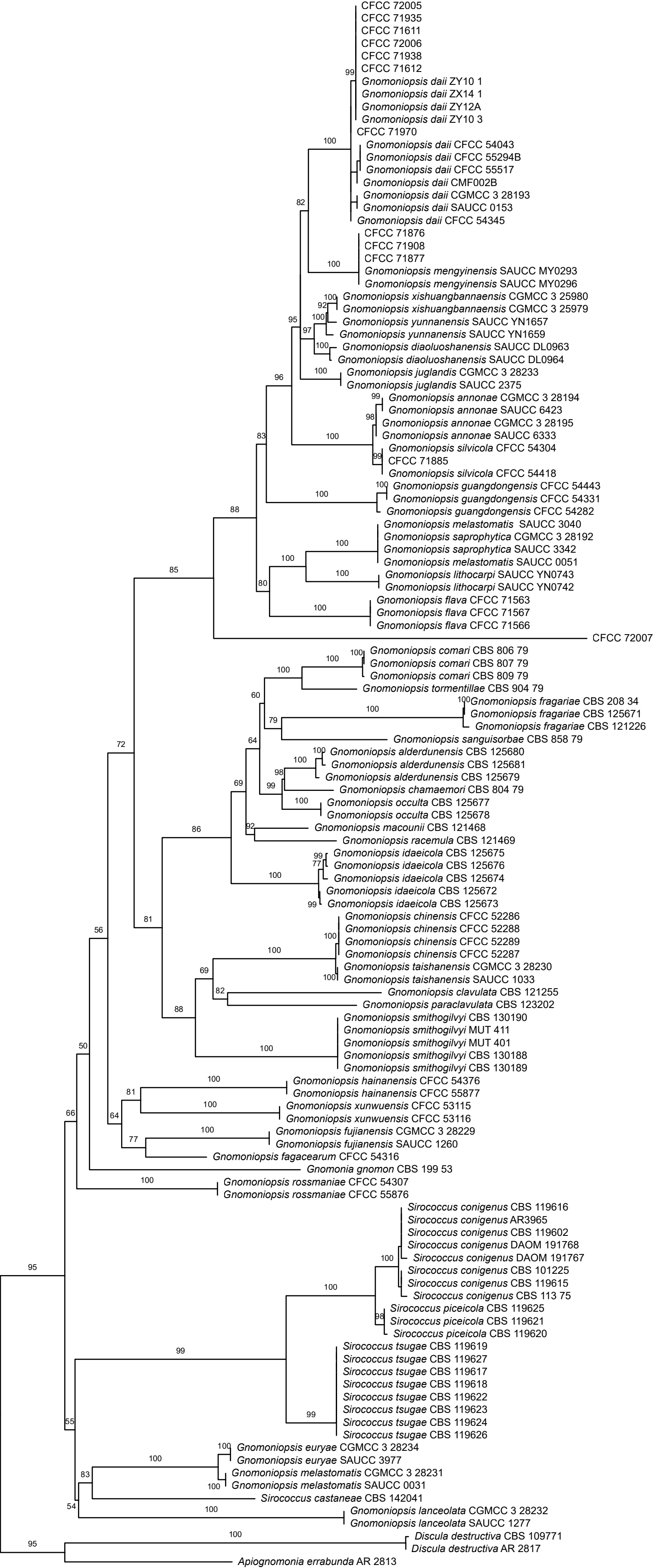

0.05

Supplement: Supplementary material 3 — Single-gene phylograms of Sirococcus [file imafungus-17-e186049-s003.zip › 186049_0R-1-A_tef_phylogeny.pdf]

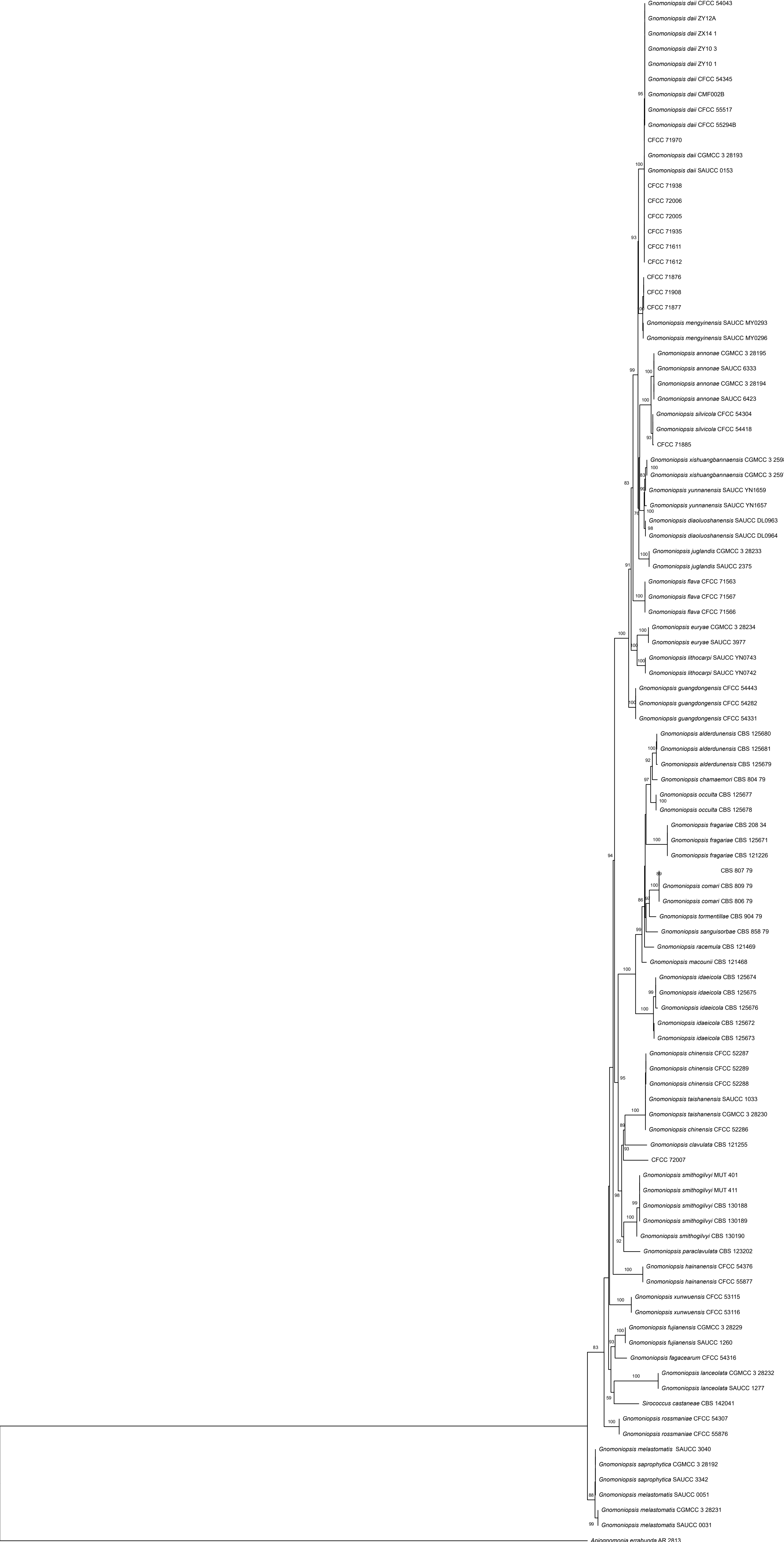

Supplement: Supplementary material 3 — Single-gene phylograms of Sirococcus [file imafungus-17-e186049-s003.zip › 186049_0R-1-A_tub_phylogeny.pdf]
